# Modelling breast cancer requires identification and correction of a critical cell lineage-dependent transduction bias

**DOI:** 10.1038/ncomms7927

**Published:** 2015-04-21

**Authors:** William C. Hines, Paul Yaswen, Mina J. Bissell

**Affiliations:** 1 Life Sciences Division, Lawrence Berkeley National Laboratory, Mailstop 977R225A, 1 Cyclotron Road, Berkeley, California 94720, USA

## Abstract

Clinically relevant human culture models are essential for developing effective therapies and exploring the biology and etiology of human cancers. Current breast tumour models, such as those from oncogenically transformed primary breast cells, produce predominantly basal-like properties, whereas the more common phenotype expressed by the vast majority of breast tumours are luminal. Reasons for this puzzling, yet important phenomenon, are not understood. We show here that luminal epithelial cells are significantly more resistant to viral transduction than their myoepithelial counterparts. We suggest that this is a significant barrier to generating luminal cell lines and experimental tumours *in vivo* and to accurate interpretation of results. We show that the resistance is due to lower affinity of luminal cells for virus attachment, which can be overcome by pretreating cells—or virus—with neuraminidase. We present an analytical method for quantifying transductional differences between cell types and an optimized protocol for transducing unsorted primary human breast cells in context.

## Abstract (web summary)

Clinical breast cancers predominantly present luminal features, but experimental models are essentially basal. Here the authors show that luminal cells are significantly less susceptible to viral transduction, and present methods to analyse and overcome the bias in heterogeneous populations.

## Graphical abstract

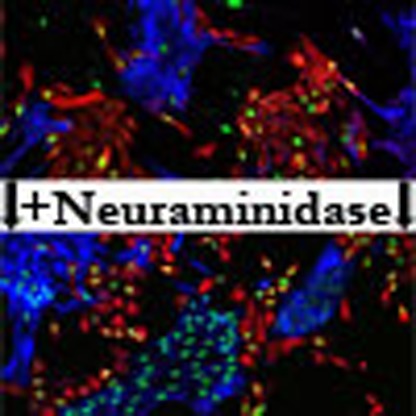

The breast is an intricate structural composition of epithelial and endothelial cells, adipocytes, fibroblasts and other immune and bone marrow derived cells, among others. Breast cancers arise from the epithelial compartment, which consists of both luminal epithelial and myoepithelial cells (LEPs and MEPs)^1^. Interactions between these cells along with other cells and extracellular molecules in the tissue microenvironment substantially influence cell physiology and tumour development, ultimately leading to tumours with distinct pathologies (reviewed in refs 2, 3, 4). Although breast cancers are complex heterogeneous entities, they fall into several molecularly defined ‘intrinsic subtypes'^5^,^6^. Most prevalent are the luminal tumours; they constitute 75–80% of breast cancer cases^7^ and characteristically express receptors for oestrogen and progesterone hormones. Whereas most of these respond well to treatment, about 30% either are—or progress to—forms that are more aggressive^8^. Learning what distinguishes this population from the rest is critical to our understanding of how to treat breast cancer patients effectively.

The answer to this question has nevertheless been hampered by the dearth of representative models of luminal cancer, including those produced by genetically engineered mice and xenografts^9^,^10^,^11^. This includes also tumours formed from existing luminal cell lines, which fail to produce key histological features of luminal breast cancers^12^. Accurate models of luminal cells and cancers are thereby needed to explore the fundamental processes specific to this cell subtype to gain a more thorough understanding of breast cancer.

Current methods for generating such models are to isolate cancer cells directly from tumours/metastases or to transform normal cells by viral transduction (for review, see refs 10, 13). Culturing luminal tumour cells from clinical samples has proven to be particularly challenging because of the difficulties adapting these cells to growth conditions and either selection of—or conversion to—basal phenotypes in culture^12^. The second option of transducing cells derived from normal tissues^14^ is well suited for studying early events in malignant transformation. Yet when the primary epithelial cells from breast reduction tissues, which contain both LEPs and MEPs, are treated with transforming viruses to produce xenografts, the outcome overwhelmingly favours the formation of squamous or basal-like tumours^15^,^16^,^17^,^18^,^19^; the reasons for this discrepancy are not known.

These findings are surprising because the data in the literature appear to be based on the assumption that epithelial cells in the breast (or other organs) will have a similar potential of being transduced. We show here that this assumption is unwarranted. When primary breast cultures are inoculated with lentivirus, the resulting transductions are heavily biased in favour of MEPs. Here, we provide a mechanism as to why this is so and describe a generalizable analytical method for comparing the lentiviral transduction efficiencies of heterogeneous cell populations. Most importantly, we provide a simple method to overcome this disparity and efficiently transduce luminal epithelial cells.

## Results

### Transduction of primary cells exposes a bias

**Figure 1:**
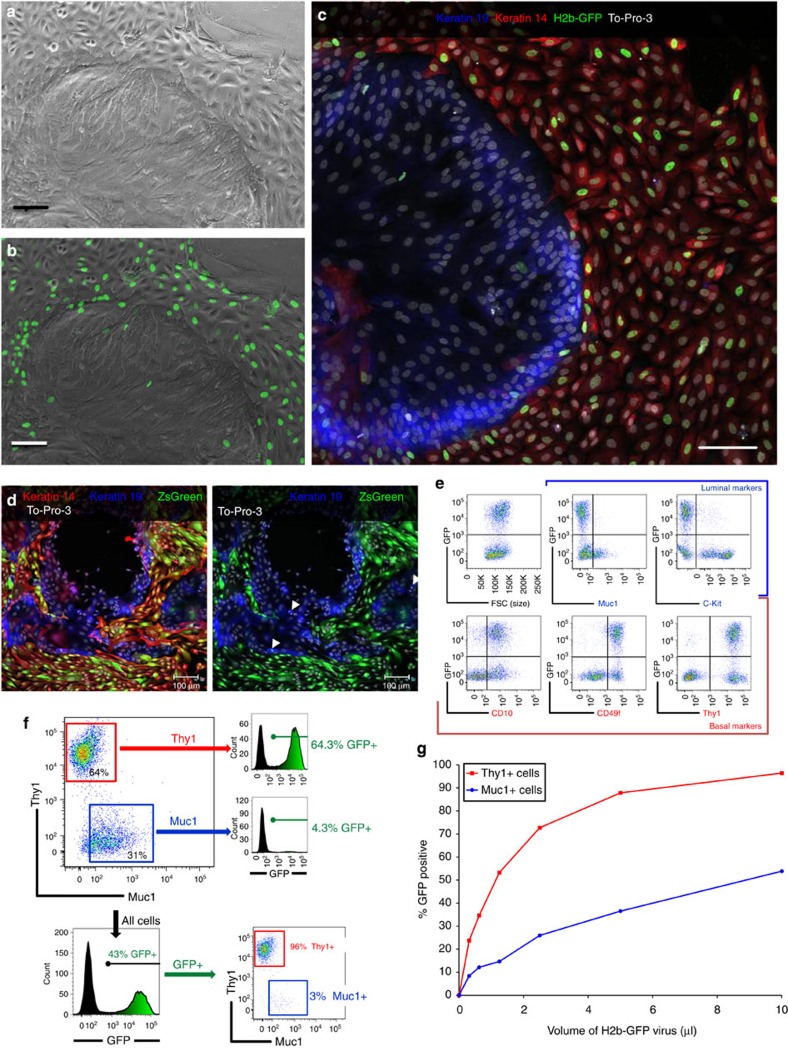
Lentiviral transduction of primary breast cells strongly favours myoepithelial cells. (**a**) Primary cell outgrowth, derived from a reduction mammoplasty tissue (RMT) from a 24-year-old woman, cultured in MCDB170 medium, and inoculated with pLenti6/CMV-H2B-GFP lentivirus (1 μg ml^−1^ polybrene). (**b**) Overlay of H2b-GFP signal. (**c**) Overlay of keratin 19 (blue) and keratin 14 (red) immunofluorescence with TO-PRO-3 nuclear counterstain (white). (**d**, left) Primary breast cells (passage 1), derived from RMT from a 34-year-old woman, transduced with pLenti6/CMV-ZsGreen lentivirus (+6 μg ml^−1^ polybrene) and immunostained as in **c**. (**d**, right) Digital removal of red keratin 14 signal; arrowheads mark 3 of the 12 k19^+^ mitotic cells (**e**) Flow cytometric characterization of primary cells, derived from RMT from a 26-year-old woman (sample N135), cultured in M87 medium and inoculated with pLenti6/CMV-H2B-GFP lentivirus. GFP in transduced cells is compared with the cell expression of lineage markers associated with luminal (Muc1, c-Kit) and basal (CD10, CD49f, Thy1) cell types. (**f**) Quantification of flow cytometry data shown in **e**. (**g**) Transduction efficiencies of first passage of N135 cells (mixed culture) inoculated with twofold serial dilutions of 1,500 × concentrated CMV-H2B-GFP lentivirus. The fraction of GFP+ cells in the MEPs and LEPs was determined by multi-parameter flow cytometry using Muc1 and Thy1 specific antibodies. The transductional bias has been observed in every (over two dozens) primary culture tested to date. (**a**–**d**) Scale bars, 100 μm.

Primary breast cultures established from reduction mammoplasty tissues contain diverse populations of cells with distinct morphologies (Fig. 1a). Continuous passaging of these cells leads to a dramatic phenotypic drift through competitive selection of cells exhibiting or acquiring a basal phenotype^10^,^13^,^20^,^21^,^22^. We therefore used only primary or first-passage cells to maintain the cellular heterogeneity of the tissue, and transduced these cultures with different fluorescent protein-encoding lentiviral vectors. The finding of a sharp delineation between transduced and untransduced cells (Fig. 1b) led us to hypothesize that viral susceptibility may be lineage dependent. This was indeed the case: staining virus-treated cultures for LEP- and MEP-specific markers (keratin 19 and 14) indicated that whereas the majority of MEPs expressed green fluorescent protein (GFP), very few LEPs were transduced (Fig. 1c). These findings were independent of the promoter–reporter combinations used (Fig. 1d), and the bias was present in both primary and secondary cultures and with all lentiviral constructs tested (Fig. 1a–d and Supplementary Table 1).

To quantify the transduction efficiencies, we used multi-parameter flow cytometry and antibodies specific for markers of LEPs (Muc1, c-Kit) and MEPs (CD10, CD49f, Thy1; Fig. 1e). In each case, viral resistance tracked with markers of the luminal phenotype, confirming the immunofluorescence data. Selection of the transduced cells led to a dramatic shift in the relative proportions of LEPs and MEPs present, as demonstrated by the analysis of GFP expression in the Thy1- and Muc1-expressing cells (Fig. 1f). We observed this bias when cultures were inoculated either as unsorted-heterogeneous or fluorescence-activated cell sorting (FACS)-purified populations, on two-dimensional substratum or in suspension; it persisted in both primary and secondary cultures at all lentiviral doses, even at high multiplicities of infection of 680 transductional units per cell (Fig. 1g, Supplementary Fig. 1 and Supplementary Table 1). The bias also did not correspond to quiescence of the LEP subpopulation or to differences in growth rate as assayed by staining of Ki-67 and incorporation of EdU (5-ethynl-2′-deoxyuridine; Fig. 1d, Supplementary Fig. 2). Moreover, a broad mechanism of resistance was indicated by the fact that lentiviruses pseudotyped with a set of envelope glycoproteins from other viral species did not increase LEP transduction efficiency (Supplementary Fig. 3). These findings provided further evidence supporting our initial observations linking viral resistance to the luminal cell lineage.

**Figure 2:**
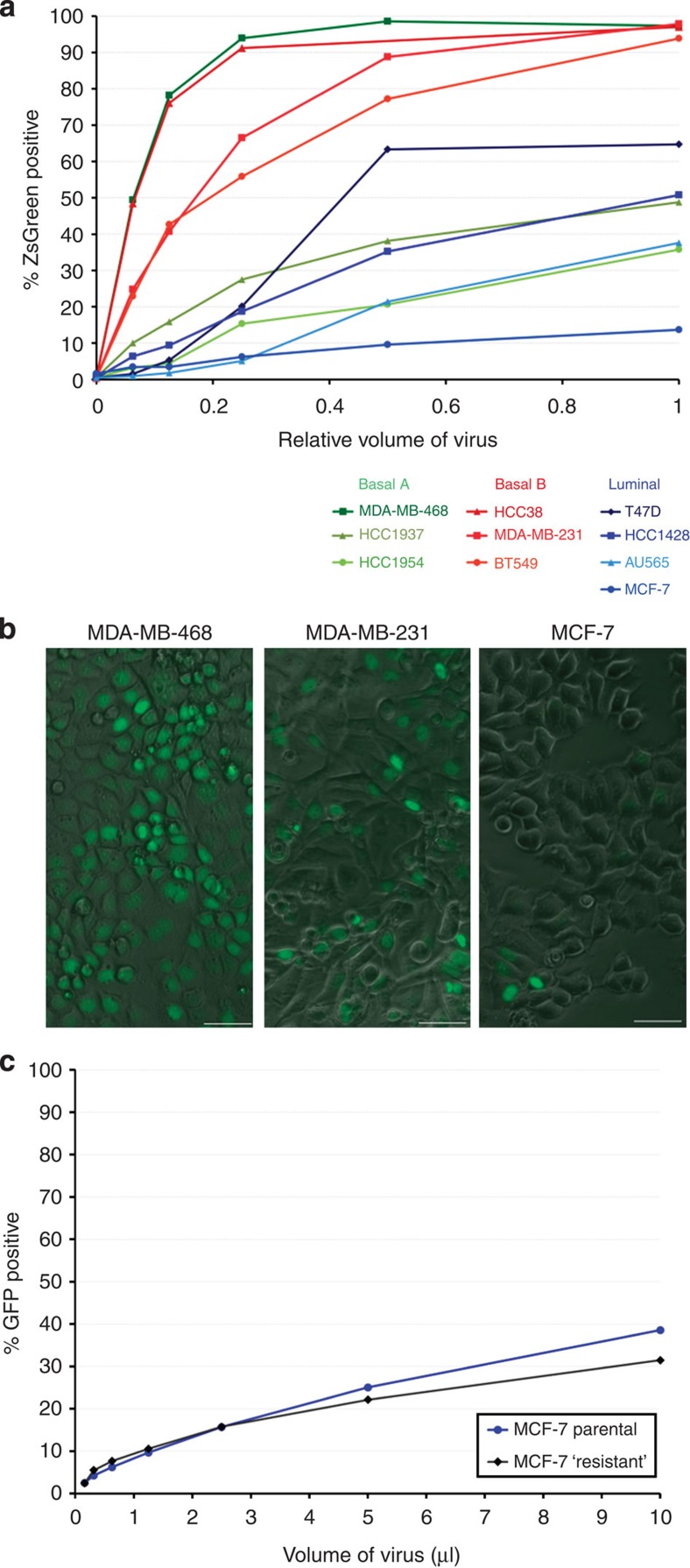
Cancer-derived cell lines also exhibit a lineage-related transductional bias. (**a**) Transductional efficiencies exhibited by 10 breast cancer cell lines, grouped by their mRNA expression profiles. Cell lines were inoculated in parallel with the identical twofold serial dilution series of pLenti6/CMV-ZsGreen lentivirus (inoculated in DMEM/10% FBS, 6 μg ml^−1^ polybrene) and analysed by FACS. (**b**) Fluorescent and phase-contrast image overlays of three representative cell lines, MDA-MB-468, MDA-MB-231 and MCF-7, inoculated with pLenti6/CMV-H2B-GFP lentivirus. (**c**) Flow cytometry analysis of MCF-7 cells inoculated with a twofold dilution series of pLenti6/CMV-H2B-GFP lentivirus (‘Parental,' blue trace). Virally resistant cells (GFP negative) were sorted by FACS from the culture inoculated with the highest dose of virus. These sorted ‘resistant' cells (black trace) were reinoculated with virus and analysed under conditions identical to the previous infection.

The above results indicated that transductional sensitivities are intrinsic properties that may be preserved in MEP- and LEP-immortalized cell lines. We thus sought a cell model that would facilitate identifying why the luminal cells were more resistant. There are just a few well-characterized non-malignant breast cell lines, such as MCF10A^23^ and HMT3522-S1 (ref. 24), but both have a predominantly basal phenotype^25^,^26^. Hence, we turned to cancer-derived cell lines classified as being ‘luminal' based on their gene expression patterns^25^. We randomly selected and measured the transduction efficiencies of four of these cell lines, along with six other basal cell lines classified as being either ‘Basal A' or ‘Basal B'^25^. A wide range of transduction efficiencies were found—from 14 to 99% cells transduced (Fig. 2a). When grouped according to class, these cell lines also formed a noticeable trend that reflected the bias observed in primary cells. Whereas the three ‘Basal B' cells were all readily transduced (94, 97, 98% ZsGreen^+^), the ‘Luminal' cell lines were significantly more resistant (14, 38, 51, 64.7% ZsGreen^+^) and the ‘Basal A' cells were divergent (36, 49, 99%, Fig. 2a). To model the transductional bias observed in primary cells, we chose the most resistant luminal cell line (MCF-7) and Basal A and Basal B cell lines with high (MDA-MB-468) and intermediate (MDA-MB-231) levels of susceptibility (Fig. 2b) for further characterization.

### Cell types are transduced similarly despite bias

To distinguish between stochastic and intrinsic transduction models, we inoculated MCF-7 cells with lentivirus and isolated the GFP-negative (resistant) population by FACS. We then reinoculated these cells using conditions identical to that of the previous infection, and compared the resulting transduction efficiencies of this ‘resistant' population with that of the parental cell line (Fig. 2c). The pattern repeated itself, as selection of the GFP-negative population provided no heritable enrichment in resistance. The nearly identical transduction efficiencies of the two populations indicated there was no intrinsically ultra-resistant or sensitive subpopulation coexisting in MCF-7 cultures. Instead, this result supported a stochastic model wherein most or all cells in the culture are equally susceptible to viral transduction, although this level of susceptibility differs among cell lines.

On the basis of this finding, we hypothesized that the processes governing cell transduction of the different cell types were similar, despite their large quantitative differences. To examine this possibility, we devised a new approach to calculate and express viral titre that reliably reflects not only the contributions of cell type, but also specific conditions to infectivity (for example, type of medium, presence of fetal bovine serum (FBS), polybrene and so on that will be used in the experiment). We coined a new term, the effective cell-transducing volume (ECTV), which we defined as the volume of a viral stock that is equivalent to a single 100% effective transduction unit when applied to a given cell population under specific experimental conditions. An advantage of using ECTV over particle-based methods is that it will more accurately predict the volume of viral stock needed to achieve a given level of transduction by taking into account the probability of cells having multiple viral integrants. The use of ECTVs circumvents the need to convert volumes of virus to a measure of viral particles, such as colony-forming units or infectious-forming units, distinguishing it from other biological methods of titration. Most importantly, however, it provided us a single metric for comparing the influences of cell type and experimental conditions to viral infectivity between cell lines, and was especially important for comparing differences among the coexisting primary cell populations. The definition, derivation and other benefits of using ECTV are explained more fully in Supplementary Notes 1 and 2.

**Figure 3:**
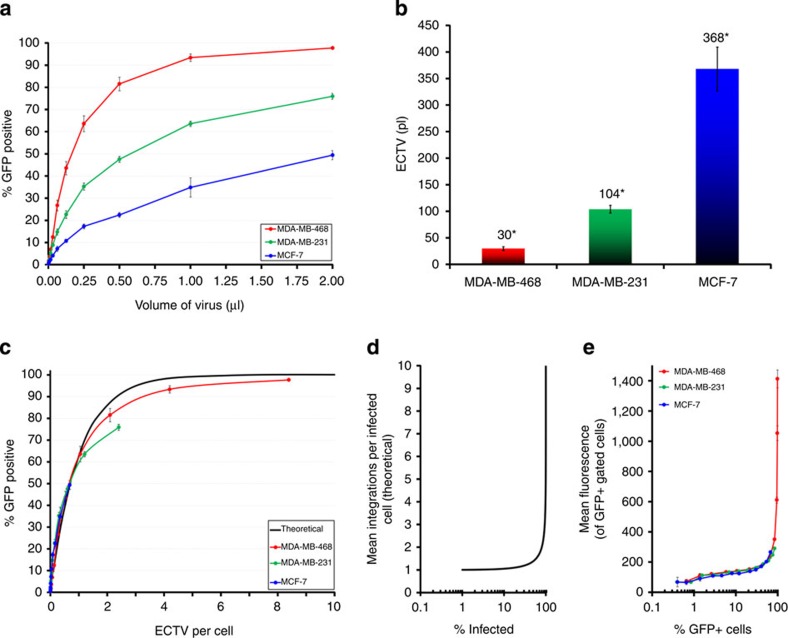
ECTVs provide insight into viral transductions. (**a**) Transduction efficiencies (±s.d.) of MDA-MB-468, MDA-MB-231 and MCF-7 cell lines inoculated with twofold serial dilutions of pLenti6/CMV-H2B-GFP lentivirus in DMEM/10% FBS. (**b**) Calculated mean ECTVs (±s.d.) for cell lines and conditions shown in **a**, reported as picoliters (pl) of viral stock; *indicates *P* value<0.01 (*t*-test for all three possible comparisons). (**c**) Fraction of transduced cells plotted with respect to the effective viral dose (ECTVs per cell); black trace indicates the theoretical fraction of transduced cells predicted by the Poisson distribution. (**d**) Poisson-predicted relationship between average number of viral integrations (per cell) that will occur at a given transductional level (% infected). (**e**) GFP levels (mean±s.d.) in transduced cells (GFP^+^ gated) compared with the fraction of all cells transduced (% GFP^+^) at each viral dose. Cells were inoculated with pLenti6/CMV-H2B-GFP lentivirus in DMEM/1% FBS.

We used this new approach to investigate the manner by which the three representative cell lines become transduced by inoculating them with serial dilutions of virus and then calculated the ECTVs for each (Fig. 3a,b). Using these values, we compared the fraction of transduced cells with the ‘effective dose' (ECTVs per cell) at each dilution, which in turn permitted a direct comparison with the theoretical values predicted by the Poisson distribution (Fig. 3c). Remarkably, we found that data from each of three cell lines fit well to the predicted values, demonstrating that the cell lines were being transduced in a similar qualitative manner (Fig. 3c), despite their quantitative differences (Fig. 3a,b). These ECTVs highlight the fundamental differences in susceptibility of each cell line to lentiviral transduction; for example, under these specific conditions, MCF-7 cells must be inoculated with over 12-fold (368/30) more virus than MDA-MB-468 cells to achieve an equivalent transduction efficiency (Fig.3b). Strikingly, the MEP and LEP subpopulations in primary mixed cultures followed the same pattern of transduction, and calculation of the ECTVs (from data presented in Fig. 1g) revealed a similar bias: 257 and 33 pl for the respective LEP and MEP subpopulations (7.8-fold difference, Supplementary Fig. 4).

When applied to the problem of viral transductions, the Poisson distribution predicts that the number of viral integrations a cell will acquire will sharply rise as the fraction of cells transduced approaches 100% (Fig. 3d, Supplementary Fig. 5). Using quantitative PCR (qPCR) to measure viral integrations in transduced (GFP^+^ sorted) MDA-MB-468 cells, we found this was indeed the case (Supplementary Fig. 6a–e). The association was reflected also in the per-cell GFP fluorescence measured by flow cytometry, which we found useful as a proxy for per-cell integrations (Supplementary Fig. 6d). Moreover, the pattern was the same for each of the three cell lines (Fig. 3e), supporting the notion that the processes governing cell transduction were similar among cell types, and that the subpopulations with increased viral susceptibility did not exist in these cultures. Nevertheless, the inherent differences in susceptibility among the different cell lines remained. Thus, to overcome the luminal cell resistance, we needed to identify the basis of these transductional differences.

### Neuraminidase enhances lentiviral transduction

**Figure 4:**
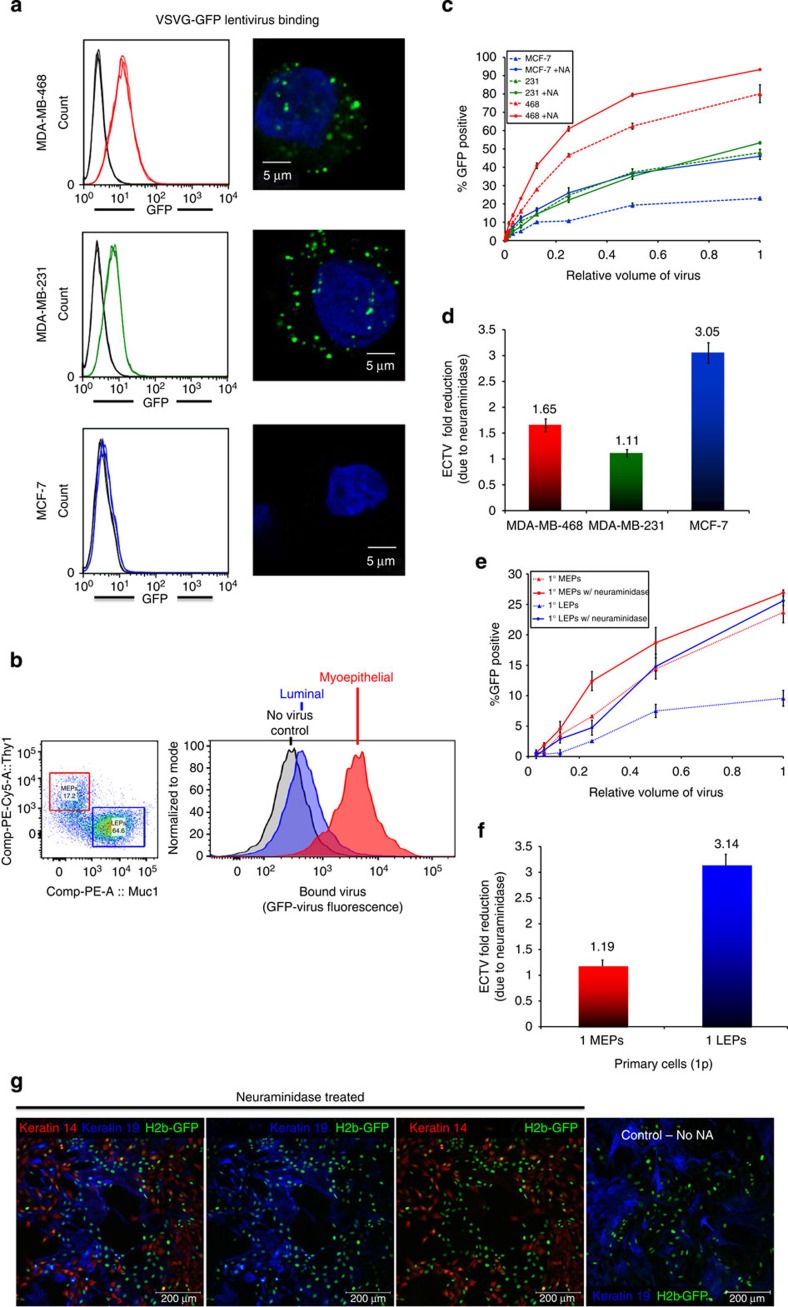
Neuraminidase enhances lentiviral transduction. (**a**, left column) Viral-binding assay: flow cytometry histograms of MDA-MB-468, MDA-MB-231 and MCF-7 cells incubated with (VSV-G-GFP) fluorescent lentiviral particles (red, green and blue traces, respectively) compared with negative controls (no virus, black traces). (**a**, right column) Confocal images of lentivirus (Green) bound to cells counterstained with DAPI (blue). (**b**) Viral binding to primary cells derived from RMT of a 32–year-old woman. (**b**, left) After incubating the cells with fluorescent lentiviral particles, the cells were stained with Muc1 and Thy1 antibodies, and analysed by flow cytometry to determine cell lineage. (**b**, right) Histograms indicating the amount of virus bound (GFP fluorescence) to luminal (LEPs, blue) and myoepithelial cells (MEPs, red). (**c**) Transduction efficiencies of the three representative cell lines, MDA-MB-468, MDA-MB-231 and MCF-7 cells (inoculated with or without 100 mU ml^−1^ neuraminidase pre-incubation) measured by flow cytometry. (**d**) ECTV reduction resulting in fold improvement of effective viral titre after neuraminidase pretreatment. (**e**) Transduction efficiencies of primary cells derived from RMT of a 31–year-old woman, inoculated with serial dilutions of pLenti6/CMV-H2B-GFP lentivirus, with—or without—neuraminidase pretreatment. Cell lineage was determined by co-staining for Thy1 (red, MEPs) and Muc1 (green, LEPs). (**f**) ECTV reduction resulting in fold improvement of effective viral titre after neuraminidase pretreatment of primary cells follow the same trend as the cell lines (**c**). (**g**) Photographs of first-passage primary cells, derived from RMT of a 20-year-old woman inoculated with pLenti6/CMV-H2B-GFP lentivirus, with—or without—neuraminidase preincubation, and stained as in Fig. 1a–d. Digital removal of keratin 14 and keratin 19 in panels 2–4 allow for better comparison to GFP signal in transduced cells.

After considering each step of viral infection and transduction, we found a critical step to be the interaction between the virus particles and cells. This was determined by constructing GFP-tagged lentiviral particles (using a GFP–VSV-G fusion construct) and incubating these fluorescent viruses in suspension with the three representative cell lines. Cellular affinity for the virus was then evaluated by confocal microscopy and flow cytometry. We found stark differences in the amount of virus bound to MCF-7 cells in comparison with the two basal cell lines (Fig. 4a). Quantification by flow cytometry showed that the affinity of the cell lines (Fig. 4a) and primary cells (Fig. 4b) to lentivirus mirrored their relative transduction efficiencies, that is, 468>231»MCF-7 and MEP»LEP (Figs 1g and 3a).

To understand the resistance of viral binding to the different cell types, we looked for a physiological explanation: there are several components in breast milk with demonstrable protective effects against a range of bacterial and viral pathogens, including Muc1 (sialomucin) and several other mucins that are expressed exclusively by luminal epithelial cells^27^. We thus hypothesized that a probable barrier to infection was attributed to cell surface glycans that are differentially expressed between cell types. We screened several glycan-modifying enzymes: α-L-Fucosidase, β-(1→3,4,6)-Galactosidase, Neuraminidase and Hyaluronidase, for their ability to alter primary cell transduction, and found both hyaluronidase and neuraminidase improved transduction efficiencies. We therefore further optimized the conditions and tested the effects of neuraminidase and hyaluronidase on cell lines and primary cells.

Pretreatment of the representative cell lines with neuraminidase before lentiviral infection improved transduction of each, having the most significant impact on MCF-7 cells (3.05-fold improvement versus 1.65 and 1.11 for MDA-MB-468 and MDA-MB-231, Fig. 4c,d, with no visible signs of toxicity or alterations in cell morphology). Similarly, pretreating primary cultures with the enzyme indeed improved the transduction efficiencies of LEPs and MEPs (Fig. 4e,f), the degree of which was remarkably similar to that observed for MCF-7 and MDA-MB-231 cell lines (compare Fig. 4f–d). Thus, with neuraminidase cell pretreatment, the respective transduction of the LEPs and MEPs was 25.6% and 26.9% at the highest viral dose, effectively equalizing transductions of these two primary cell populations (Fig. 4e).

After determining that we could dramatically alter the ratio of transduced cell types by pretreating the cells with neuraminidase, we wondered if pretreating virus with the enzyme would have a similar effect, or any at all, on the amount and types of cells transduced. Remarkably, it did. Virus treated with different concentrations of neuraminidase (20, 200 and 2,000 mU ml^−1^) incubated at two different temperatures (22° or 37 °C) improved the overall transduction of cells, as well as the ratio of LEPs to MEPs transduced, at all doses and temperatures tested (Supplementary Fig. 7). Virus incubated with 200 mU ml^−1^ neuraminidase at 37 °C, for example, improved the overall transduction efficiency from 2.57 to 18.23%, while reducing the transduced MEP:LEP ratio from 5.19 to 1.85. In viral-binding experiments, untreated virus again demonstrated a notably low affinity to LEPs, whereas virus treated with neuraminidase had a noticeably improved affinity to both primary cell types (Supplementary Fig. 8). This simple treatment of virus thus had a dramatic effect by improving the overall effective viral titre while correcting for the biased transduction between primary cell subpopulations. We found it to be easily performed after virus preparation, which circumvented the need of more lengthy cellular treatments and any unintended consequences that may have. Because of the lower volumes involved, it required also much less enzyme, reducing costs while providing similar results.

To characterize the practical application and reproducibility of our method, we tested on different primary cultures the individual and combined effects of two enzymatic pretreatments: (a) treating cells with hyaluronidase and (b) treating virus with neuraminidase. Over the course of several months, using multiple batches of virus (required for the many treatments and replicates) and independent primary cultures derived from tissues of six different subjects, we explored the individual and combined effects of these optimized treatments and found a strikingly reproducible pattern they had on narrowing (and widening) the divide between transduced MEPs and LEPs (Supplementary Fig. 9). As revealed by the transduced MEP:LEP ratios, untreated controls, consistent with our prior findings, always exhibited a bias in favour of MEPS. The degree of the bias expectedly varied among the different primary cultures, but was internally reproducible among replicate experiments, ranging from as low as 1.6-fold to as high as 4.8-fold under these conditions, and extending as high as 13.2-fold in hyaluronidase-treated cells. Although treating the cells with hyaluronidase on average led to a 63% higher fraction of cells transduced (1.63±0.59-fold), the impact on the cell types was uneven, often improving transduction of the MEPs more than the LEPs, producing an even larger bias in six out of seven experiments. Neuraminidase, however, when used to pretreat the virus before infection, reduced the bias every time (seven out of seven), by an average of 42% (0.58±0.15-fold difference in MEP:LEP). Whereas combining the two treatments (that is, cells with hyaluronidase and virus with neuraminidase) led to higher transduction efficiencies in five out of seven experiments, it resulted in slightly higher MEP:LEP ratios compared with infections using treated virus alone (2.51±0.68 versus 1.67±0.60, Supplementary Fig. 9). Therefore, we find the best method to reduce the bias between MEPs and LEPs is to use neuraminidase-treated virus.

### Creation of extended lifespan luminal cells and cell lines

Knowledge of the transductional bias and the ability to efficiently transduce primary luminal cells has enabled us to create extended lifespan cultures of LEPs that have retained their luminal phenotype for over four months in culture (20 passages, Supplementary Fig. 10). To generate these cell lines, we constructed a lentivirus encoding the SV40 early region (SV40er) and, using neuraminidase treatment, transduced primary cultures with either SV40er or H2b-GFP (control) lentiviruses, then sorted the transduced cells into LEP (Muc1^+^) and MEP (Thy1^+^) fractions. MEP cultures transduced with either SV40er or H2b-GFP grew continuously for more than 20 passages and maintained a basal phenotype (measured by K14, Thy1 and p63 staining). Whereas LEP control cells (H2b-GFP and uninfected) became senescent after the fourth passage, LEPs transduced with SV40er did not lag in their growth, and have maintained their luminal phenotype, measured by K18 and Muc1, for more than 20 passages (Supplementary Fig. 10). These results clarify that it is transduction efficiency rather than any selective or ‘differentiation-inducing property' of the SV40 early region that determines the subclasses of extended lifespan cultures obtained.

## Discussion

Cell lines created through carcinogen or oncogene exposure of cultured breast cells are essentially phenotypically ‘basal.' The reasons for this proclivity have been puzzling, but this predisposition nonetheless has resulted in a dearth of representative models of luminal breast cancer and uncertainty regarding the relevance of existing oncogenic models to the processes that induce clinical breast cancers. Here, we set out to determine the biology behind this consequential discrepancy, and to find measures that would rectify this imbalance.

Analysis of primary tissues transduced with lentiviruses led us to the discovery that regardless of the specific composition of the vector or the encoded genes, there is a substantial transductional bias in heterogeneous populations of breast cells. The finding and characterization of this bias is the single-most important aspect of the work presented here; however, nearly equally important is the identification of techniques that effectively correct this bias. We describe also a method of measurement (that is, ECTV) that can be easily and productively used to more accurately predict the volume of viral stock needed to achieve a given level of transduction. This method provides a single metric for considering viral infections and comparing obstacles that influence viral infectivity of luminal and myoepithelial cells (LEPs and MEPs) of the human breast, but which can be applied also to other tissues and cancers.

Directed oncogenic transformation of primary cells requires viral vectors for delivery of the required genes^28^. An attractive feature of lentiviral vectors is their rare ability to transduce quiescent cells, thereby avoiding yet another well-characterized selection bias, something that oncoretroviruses, such as MLV, cannot do. Consequently, lentiviruses have become the vector of choice in the field, particularly when targeting stem cells or other quiescent cell types^29^,^30^. We discovered that breast LEPs are significantly more resistant to lentiviral (or other viral) transduction than their MEP counterparts (Fig. 1). This bias was present in normal primary cells and established cell lines, and was independent of cell passage, growth rate, media, presence of polybrene, infection in suspension or specific characteristics of the viral constructs, such as the promoter, gene product or viral pseudotype (Fig. 1; Supplementary Figs 1 and 3)

We discovered that despite the substantial resistance of LEPs to lentivirus, resistance to infection is not absolute; rather the probability of LEPs becoming transduced is much lower than MEPs. This could either be because the susceptibility is intrinsic, such that there are fewer cells in the luminal compartment that are able to be transduced. Or each of the luminal cells has the same potential of being transduced, but inherent differences between luminal and basal cells exist and produce the observed transductional bias. Our data support the latter.

We found the absolute number of LEPs capable of being transduced is not fixed; using higher doses of concentrated virus in serial dilution experiments led to higher transduction efficiencies (Fig. 1g). However, regardless of the viral dose, the bias between LEPs and MEPs always remained. We show also that uninfected, ‘resistant' cells from one round of lentiviral exposure were no more resistant to subsequent exposure than the unenriched parental population from which they were derived (Fig. 2c). Most important, however, is our demonstration that the data from both luminal and basal cells—whether primary or cell lines—fit to a Poisson model of infection, demonstrating that these cells are transduced in a similar qualitative manner, despite their large quantitative differences.

The need to compare transductions of different cells simultaneously to levels predicted by the Poisson distribution led to the development of a new means to calculate viral titre, which we coined the ECTV. This is defined as the volume of virus equivalent to a single theoretical ‘transduction unit' and is dependent on the specific cell type and experimental conditions used, which emphasizes the importance of each to viral transduction. ECTV calculation incorporates predictions of the Poisson distribution and thus more reliably predicts the amount of viral stock needed to achieve a given level of transduction (Supplementary Notes 1 and 2; Fig. 3). Direct quantitative comparisons of ECTV for different cell lines led us to search for the probabilistic basis of the transductional bias as we considered each step of the viral infection process.

This turning point in our study clarified a distinction between the two major cell types in the breast, and pointed to a possible mechanism by which LEPs and MEPs could differ in resistance. We traced the source of the variability to the cell surface and showed luminal cells to be relatively deficient in their ability to bind lentivirus (Fig. 4a). This led us to consider the glycans, sugar moieties that coat the cells and play key roles in the infection process of many different viral species. Ultimately, we found that neuraminidase treatment of the cells significantly improved lentiviral transduction, more so for LEPs than MEPs, thus effectively balancing transduction of these two populations. Arcasoy *et al*.^31^ showed more than a decade ago that the inhibition of adenoviral infection of MDCK cells by Muc1 and other sialoglycoconjugates could be improved by pretreating the cells with neuraminidase before infection. Whether the mechanism of this effect is the same between adenovirus and lentivirus, or even MDCK canine cells and primary human breast cells, remains a mystery. However, we find that to obtain a balancing effect in primary breast cells, treatment of the cells is not necessarily required: treating virus alone significantly improves the ratio of LEPs to MEPs transduced. Notably, hyaluronidase treatment of cells also improved transductions, but often led to an even greater bias between cell types.

Some researchers use hyaluronidase along with collagenase when digesting tissues; these conditions may thus cause an even higher transductional bias than what we report using tissues digested with collagenase alone. It is our experience that even slight differences in digest protocols can have dramatic and misleading consequences^32^. Knowledge of the transductional imbalance, along with the ability to overcome it, will likely provide for a higher level of reproducibility.

There are profound implications for the ability to balance lentiviral transductions, and we highlight some in the context of developing culture models of cancer: The first is that developing luminal cell lines and models of luminal cancer have been woefully difficult and yet crucial for understanding three-fourth of all breast cancers. We believe the bias described here has been a significant barrier to developing such models. Current models of transformation rely on multiple viral transductions, such that the bias, which is already quite large for a single vector, expands by compounding the probabilities with each vector added. One example of this is the work of Kuperwasser and co-workers^18^, who employed an immunomagnetic enrichment strategy before viral transduction with oncogene combinations. Consistent with our findings, these authors noted that the transformation of unsorted populations resulted in tumours with primarily basal features, whereas oncogenic transduction of luminal marker-enriched cell population resulted in tumours with partial luminal characteristics. The reason behind the observation was not explored. We have now, after controlling for this bias, succeeded in passaging SV40er transduced luminal cells for more than 20 passages where they retain their luminal characteristics, effectively creating missing models of luminal breast cancers, but most importantly clearing a path for future developments. Relative contributions of starting cell subtypes and oncogene combinations, as well as microenvironmental factors, to the range of individual features expressed by resulting tumours are important topics for future research that will be enabled by more uniform viral transduction efficiencies made possible by the techniques presented herein. We submit that these concepts and procedures open an opportunity to study not only breast tumour heterogeneity, but would be applicable also to a range of other organs and tumours.

## Methods

### Breast tissues and primary cultures

Breast tissues from reduction mammoplasties were obtained from the Cooperative Human Tissue Network, a programme funded by the National Cancer Institute. All specimens were collected with patient consent and were reported negative for proliferative breast disease by board-certified pathologists. Use of these anonymous samples was granted exemption status by the University of California at Berkeley Institutional Review Board according to the Code of Federal Regulations 45 CFR 46.101. On receipt, the tissues were minced and treated with 0.1% collagenase I (Gibco/Invitrogen) for 12–18 h in Dulbecco's Modified Eagle Medium containing 100 U ml^−1^ penicillin, 100 μg ml^−1^ streptomycin and 100 μg ml^−1^ Normocin (Invivogen, San Diego, CA) with gentle agitation^32^. The resulting divested tissue fragments (organoids) were collected by centrifugation (100*g* × 2 min) and either archived in liquid nitrogen (90% FBS+10% dimethylsulphoxide) or immediately placed into culture using serum-free MCDB170 (Lonza)^33^ or M87 (M87+CT+X) minimal serum (0.25% FBS) medium^34^, as indicated in the figure legends.

### Cell lines

MDA-MB-231, HCC38, BT549, T47D, HCC1428, AU565, MCF-7, MDA-MB-468, HCC1937 and HCC1954 breast cancer-derived cell lines were obtained directly from the American Type Culture Collection (ATCC). Media and culture conditions are provided in Supplementary Table 2; any deviations from these conditions are noted within figure legends. ATCC designation and passage number are provided in the Supplementary Methods (Supplementary Table 2).

### Reagents and antibodies

Anti-CD49f, c-Kit and EpCam antibodies were obtained from BioLegend (San Diego, CA); Anti-CD10, Muc1 and Thy1 antibodies were obtained from BD Biosciences (San Jose, CA); and anti-keratin 14 and keratin 19 antibodies were purchased from Neomarkers/ThermoScientific (Fremont, CA). Detailed information on the clones and conjugates are provided in Supplementary Table 3. Muc1 antibody was custom labelled using the PacificBlue Antibody Labeling Kit (Invitrogen, Carlsbad, CA). Polybrene/hexadimethrine bromide (H9268), α-L-Fucosidase from bovine kidney (F5884), β-(1→3,4,6)-Galactosidase (G1288), Hyaluronidase (H3506) and neuraminidase (type III) from *Vibrio cholera* (N7885) were purchased from Sigma-Aldrich (St Louis, MO).

### Lentiviral constructs

Lentiviruses used in this study (pLenti6, Invitrogen) are derived from a third-generation human immunodeficiency virus -1-based self-inactivating lentiviral vector^35^. Lentiviral transfer vectors were constructed using the modular MultiSite Gateway cloning technology (Invitrogen) to generate pLenti6/UbC-EGFP, pLenti6/CMV-ZsGreen, pLenti6/CMV-H2B-GFP, pLenti6/UbC-mCherry and pLenti/CMV-SV40er. Detailed cloning information is provided in the Supplementary Materials.

### Lentivirus production and titration

To prepare VSV-G-pseudotyped lentivirus particles, twenty 150-mm culture dishes, containing 80–85% confluent HEK293FT cells, were calcium phosphate transfected with an equimolar mix of plasmids (57.5 μg per dish), containing the desired pLenti6 transfer vector and three lentiviral packaging plasmids: pLP1 (gag/pol), pLP2 (Rev) and pLP/VSV-G (VSV-G, Invitrogen). Supernatant was collected at 48 and 72 h post transfection and filtered through a 0.4-μm Nalgene filtration unit. Lentivirus particles in this 600 ml of filtrate were concentrated by sequential rounds of ultracentrifugation (100,000*g* for 90 min) through a 20% sucrose/PBS cushion. The final pellet was dissolved in 400 μl of Hank's balanced salt solution and vortexed in a foam microtube holder for 30 min at room temperature. The 1,500 × concentrated virus was cleared of sediment by centrifuging at 13,000*g* for 5 min. If performed, a fraction of the lentivirus preparation was treated with neuraminidase at this stage, the specific details of which are provided in the figure legends. Controls, that is, untreated virus, were incubated in parallel under identical conditions. Virus was stored at −80 °C in either 10 or 20 μl aliquots before titration/use. Physical titre was determined by p24 enzyme-linked immunosorbent assay^36^ using plates and standards from the National Cancer Institute AIDS and Cancer Virus Program (Frederick, MD). Vector yield of VSV-G-pseudotyped lentivirus ranged between 2.0 × 10^5^ and 3.1 × 10^5^ ng of p24 per ml of concentrated virus stock, an average of 2.72 × 10^10^ TU ml^−1^. Biological activity of the virus was determined by inoculating the three cell lines (MCF-7, MDA-MB-231 and MDA-MB-468) with 2 × dilution series of lentivirus, and measuring the fraction of fluorescent cells by flow cytometry 3 days after inoculation. Calculation of ECTVs is described in the body of the manuscript and detailed in Supplementary Note 1. Alternate lentiviral pseudotypes were prepared by substituting the VSV-G-encoding plasmid with those encoding glycoproteins derived from either Rabies (Addgene 15785), Mokola (Addgene 15811), LCMV (Addgene plasmids 15793 and 15796), MMLV (Addgene 15799)^37^; Ebola (pEZGP and EboZ delta O), a gift of Dr David Sanders^38^; or Baculovirus (gp64/PCDNA3.1), generously provided by Dr Joshua Zimmerberg^39^. GFP-labelled virions used in the binding assay (Ubc-mCherry (GFP–VSV-G)) were similarly produced by replacing the VSV-G-encoding plasmid for GFP–VSV-G (Addgene 11912)^40^.

### Cell inoculation/infection

Primary cells (typically grown for 5–7 days) in 24-well dishes, were inoculated overnight in 250 μl medium containing desired amount of virus, typically 1–10 μl of a 1,500 × concentrated stock. Cells pretreated with neuraminidase received a 4-h incubation at 4 °C with 200 mU ml^−1^ neuraminidase diluted in growth medium (M87) and were thoroughly rinsed before adding virus-containing medium. All infections were performed at 37 °C overnight (at least 15 h). The following morning, the virus-containing medium was removed and refreshed with 500 μl growth medium and the cells were cultured for an additional three days to allow for GFP expression before analysis by microscopy or flow cytometry. Serial dilution experiments were similarly performed using either a 24-well or a 96-well format. For 24-well dishes, 50,000 cells were seeded into each well, allowed to attach overnight and incubated in 250 μl medium containing 2 × dilutions of lentivirus. For 96-well format, 8,000 cells were seeded and infected in 50 μl volume. Specific details to each experiment are contained in the figure legends. Polybrene did not improve the transductional bias and we do not recommended using it with primary cells because it alone induced dramatic morphological changes in the cells at concentrations as low as 5 μg ml^−1^ (Supplementary Fig. 11).

### Immunofluorescence

Immunofluorescence was performed on monolayer cell cultures fixed with 4% paraformaldehyde for 5 min at 23 °C, and then treated with 4% formaldehyde/0.1% saponin (BD Cytofix/Cytoperm kit) for 15 min at 4 °C. The cells were subsequently incubated for 20 min. in wash buffer (0.1% saponin/1% FBS in PBS), and incubated with keratin 14 (rabbit polyclonal, Thermo/labvision) and keratin 19 (mouse clone A53-B/A2.26, Neomarkers) antibodies diluted 1:200 (1 μg ml^−1^) in wash buffer for 1 h at 37 °C. Following the primary antibody incubation, the cells were washed and incubated with anti-mouse and anti-rabbit secondary antibodies, respectively, conjugated with Alexafluor 405 and Alexfluor 594 (Invitrogen), diluted 1:400. Nuclei were stained by incubating cells in 1 μM To-Pro-3 iodide (Invitrogen). Four-colour images were captured using a Zeiss LSM710 confocal microscope and processed using Zen Software (Zeiss, version 2009).

### Virus-binding assay

Lentivirus binding analysis was performed as previously described^41^. In brief, MDA-MB-468, MDA-MB-231 and MCF-7 cells were dissociated with trypsin, rinsed in PBS/2% FBS and filtered through a 40-μm cell strainer. Primary cells were dissociated similarly, but were first treated with non-enzymatic dissociation solution (Sigma# C1419) to reduce the amount of trypsin required, which was inactivated by 0.1% w/v soybean trypsin inhibitor (Sigma# T9128). To 1 × 10^5^ cells, 10 μl of a 1,500 × concentrated lentivirus UbC-mCherry(GFP–VSV-G) or 10 μl PBS (negative control) was added, and the cells were incubated at 4 °C in the dark, with gentle rocking for 2 h. Afterwards, the cells were washed once with PBS and analysed by flow cytometry (BD FACS Calibur). Remaining cells were fixed in 2% paraformaldehyde, counterstained with DAPI (4′,6-diamidino-2-phenylindole), mounted to slides with Fluormount-G (Southern Biotech; Birmingham, AL) and imaged using a Zeiss LSM710 confocal microscope.

### qPCR viral integration assay

To measure lentiviral integrations in the host cell genome, we transduced MDA-MB-468 cells (grown in DMEM/10% FBS) with twelve 2 × serial dilutions of CMV-H2b-GFP virus, diluted in M87 medium. After overnight incublation, the medium was refreshed with regular growth medium, DMEM/10% FBS. At 3 days post innoculation, the cells were photographed, dissociated and the GFP^+^ fractions were measured and FACS sorted into either 6, 24, 48 or 96-well dishes (dependent on transduction efficiency per cell yield). After expansion in culture for 1 week, DNA was isolated (DNEasy columns, Qiagen) from cultures derived from dilutions 1–9 (which had accumulated enough cells at that time). Viral integrations in genomic DNA were measured by qPCR using primers specific to the lentiviral GAG sequence (For: 5′- AGG GAG CTA GAA CGA TTC GCA GTT -3′, Rev: 5′- TCT GAT CCT GTC TGA AGG GAT GGT -3′), Lentiviral gene dose was normalized to the single copy gene, albumin^42^, (FOR: 5′- TGT AGA GAA GTG CTG CAA GGC TGA -3′, REV:5′- TGT CCC ACA TGT ACA AAG CCT CCT -3′). PCR reactions (45 cycles: 95 °C × 15 s, 60 °C × 60 s) were performed in quadruplicate and quantified using the ddCT method; error was propagated using the square root of the sum of squares method and values are expressed as a percentage of albumin.

### Flow cytometry and FACS

Lentiviral transductions of primary cells were analysed by multi-parameter flow cytometry at 72–96 h post inoculation by first dissociating the cells to single-cell suspensions with trypsin, and filtering them through 40-μm nylon mesh cell strainers (BD Biosciences). Cells were rinsed twice with PBS/2% FBS and incubated with conjugated antibodies for 30 min at 4 °C. Flow cytometry data (typically 20,000 gated events per sample) were collected. Cells were sorted using a BD FACS Vantage cytometer (FACSDIVA software, version 5.0.3). Doublets were excluded by forward scatter (height) vs. side scatter (width) gating. Compensation was determined using compensation beads custom-labelled with each fluorophor (APC anti-mouse bead kit (Molecular Probes/Invitrogen). Negative controls consisted of unlabelled beads and cells incubated with isotype control antibodies conjugated to PE, APC, PE/Cy5, FITC (BD Biosciences); PE/Cy7, APC/Cy7 (Biolegend); and Pacific Blue (Invitrogen). Serial dilution experiments were collected on a BD FACS Calibur with robotic high throughput sampler (HTS) attachment (5,000 events per sample) in a 96-well format. All FACS data were analysed using Flowjo software (version 7.6.3, Tree Star Inc.).

### Statistical analysis

Statistical analysis was performed using JMP 7 statistical software (SAS Institute). Error for the quotient ‘fold difference in ECTV,' was calculated using standard deviations (s.d.) of triplicate parallel infections to determine per cent relative error and propagated using the square root of the sum of squares method. In all other cases, error bars indicate the s.d. of multiple biological replicates.

## Author contributions

W.C.H. and M.J.B. were responsible for the study conception and design, methodology development and analysis and interpretation of data; W.C.H., P.Y. and M.J.B. were responsible for the acquisition of data (provided reagents, acquired patient samples, provided facilities); W.C.H., P.Y. and M.J.B. were responsible for writing, review and revision of the manuscript.

## Additional information

**How to cite this article:** Hines, W. C. *et al*. Modelling breast cancer requires identification and correction of a critical cell lineage-dependent transduction bias. *Nat. Commun.* 6:6927 doi: 10.1038/ncomms7927 (2015).

## Supplementary Material

Supplementary Information Supplementary Figures 1-11, Supplementary Tables 1-4, Supplementary Notes 1-2, Supplementary Methods and Supplementary References

## References

[b1] Hoda S. A., Brogi E., Koerner F. & Rosen P. P. Rosen's Breast Pathology Lippincott Williams & Wilkins (2014).

[b2] Tarin D. Clinical and biological implications of the tumor microenvironment. Cancer Microenviron. 5, 95–112 (2012). 22782446 10.1007/s12307-012-0099-6 PMC3399064

[b3] Bissell M. J. & Radisky D. Putting tumours in context. Nat. Rev. Cancer 1, 46–54 (2001). 11900251 10.1038/35094059 PMC2975572

[b4] Bissell M. J. & Hines W. C. Why don't we get more cancer? A proposed role of the microenvironment in restraining cancer progression. Nat. Med. 17, 320–329 (2011). 21383745 10.1038/nm.2328 PMC3569482

[b5] Perou C. M. . Molecular portraits of human breast tumours. Nature 406, 747–752 (2000). 10963602 10.1038/35021093

[b6] Cancer Genome Atlas Network. Comprehensive molecular portraits of human breast tumours. Nature 490, 61–70 (2012). 23000897 10.1038/nature11412 PMC3465532

[b7] O'Brien K. M. . Intrinsic breast tumor subtypes, race, and long-term survival in the Carolina Breast Cancer Study. Clin. Cancer Res. 16, 6100–6110 (2010). 21169259 10.1158/1078-0432.CCR-10-1533 PMC3029098

[b8] Massarweh S. & Schiff R. Unraveling the mechanisms of endocrine resistance in breast cancer: new therapeutic opportunities. Clin. Cancer Res. 13, 1950–1954 (2007). 17404074 10.1158/1078-0432.CCR-06-2540

[b9] Vargo-Gogola T. & Rosen J. M. Modelling breast cancer: one size does not fit all. Nat. Rev. Cancer 7, 659–672 (2007). 17721431 10.1038/nrc2193

[b10] Petersen O. W. & Polyak K. Stem cells in the human breast. Cold Spring Harb. Perspect. Biol. 2, a003160 (2010). 20452965 10.1101/cshperspect.a003160 PMC2857168

[b11] Borowsky A. D. Choosing a mouse model: experimental biology in context--the utility and limitations of mouse models of breast cancer. Cold Spring Harb. Perspect. Biol. 3, a009670 (2011). 21646376 10.1101/cshperspect.a009670 PMC3181037

[b12] Holliday D. L. & Speirs V. Choosing the right cell line for breast cancer research. Breast Cancer Res. 13, 215 (2011). 21884641 10.1186/bcr2889 PMC3236329

[b13] Dimri G., Band H. & Band V. Mammary epithelial cell transformation: insights from cell culture and mouse models. Breast Cancer Res. 7, 171–179 (2005). 15987472 10.1186/bcr1275 PMC1175079

[b14] Bartek J. . Efficient immortalization of luminal epithelial cells from human mammary gland by introduction of simian virus 40 large tumor antigen with a recombinant retrovirus. Proc. Natl Acad. Sci. USA 88, 3520–3524 (1991). 1708884 10.1073/pnas.88.9.3520 PMC51483

[b15] Elenbaas B. . Human breast cancer cells generated by oncogenic transformation of primary mammary epithelial cells. Genes Dev. 15, 50–65 (2001). 11156605 10.1101/gad.828901 PMC312602

[b16] Ince T. A. . Transformation of different human breast epithelial cell types leads to distinct tumor phenotypes. Cancer Cell 12, 160–170 (2007). 17692807 10.1016/j.ccr.2007.06.013

[b17] Wu M. . Dissecting genetic requirements of human breast tumorigenesis in a tissue transgenic model of human breast cancer in mice. Proc. Natl Acad. Sci. USA 106, 7022–7027 (2009). 19369208 10.1073/pnas.0811785106 PMC2669443

[b18] Keller P. J. . Defining the cellular precursors to human breast cancer. Proc. Natl Acad. Sci. USA 109, 2772–2777 (2012). 21940501 10.1073/pnas.1017626108 PMC3286919

[b19] Zhao J. J. . Human mammary epithelial cell transformation through the activation of phosphatidylinositol 3-kinase. Cancer Cell 3, 483–495 (2003). 12781366 10.1016/s1535-6108(03)00088-6

[b20] Taylor-Papadimitriou J. . Keratin expression in human mammary epithelial cells cultured from normal and malignant tissue: relation to in vivo phenotypes and influence of medium. J. Cell Sci. 94, 403–413 (1989). 2483723 10.1242/jcs.94.3.403

[b21] Ethier S. P., Mahacek M. L., Gullick W. J., Frank T. S. & Weber B. L. Differential isolation of normal luminal mammary epithelial cells and breast cancer cells from primary and metastatic sites using selective media. Cancer Res. 53, 627–635 (1993). 8425198

[b22] Pechoux C., Gudjonsson T., Ronnov-Jessen L., Bissell M. J. & Petersen O. W. Human mammary luminal epithelial cells contain progenitors to myoepithelial cells. Dev. Biol. 206, 88–99 (1999). 9918697 10.1006/dbio.1998.9133

[b23] Soule H. D. . Isolation and characterization of a spontaneously immortalized human breast epithelial cell line, MCF-10. Cancer Res. 50, 6075–6086 (1990). 1975513

[b24] Briand P., Petersen O. W. & Van Deurs B. A new diploid nontumorigenic human breast epithelial cell line isolated and propagated in chemically defined medium. In Vitro Cell. Dev. Biol. 23, 181–188 (1987). 3558253 10.1007/BF02623578

[b25] Neve R. M. . A collection of breast cancer cell lines for the study of functionally distinct cancer subtypes. Cancer Cell 10, 515–527 (2006). 17157791 10.1016/j.ccr.2006.10.008 PMC2730521

[b26] Kenny P. A. . The morphologies of breast cancer cell lines in three-dimensional assays correlate with their profiles of gene expression. Mol. Oncol. 1, 84–96 (2007). 18516279 10.1016/j.molonc.2007.02.004 PMC2391005

[b27] Schroten H. . Inhibition of adhesion of S-fimbriated Escherichia coli to buccal epithelial cells by human milk fat globule membrane components: a novel aspect of the protective function of mucins in the nonimmunoglobulin fraction. Infect. Immun. 60, 2893–2899 (1992). 1377184 10.1128/iai.60.7.2893-2899.1992 PMC257251

[b28] Hahn W. C. . Creation of human tumour cells with defined genetic elements. Nature 400, 464–468 (1999). 10440377 10.1038/22780

[b29] Akkina R. K. . High-efficiency gene transfer into CD34+ cells with a human immunodeficiency virus type 1-based retroviral vector pseudotyped with vesicular stomatitis virus envelope glycoprotein G. J. Virol. 70, 2581–2585 (1996). 8642689 10.1128/jvi.70.4.2581-2585.1996 PMC190105

[b30] Welm B. E., Dijkgraaf G. J., Bledau A. S., Welm A. L. & Werb Z. Lentiviral transduction of mammary stem cells for analysis of gene function during development and cancer. Cell Stem Cell 2, 90–102 (2008). 18371425 10.1016/j.stem.2007.10.002 PMC2276651

[b31] Arcasoy S. M. . MUC1 and other sialoglycoconjugates inhibit adenovirus-mediated gene transfer to epithelial cells. Am. J. Respir. Cell Mol. Biol. 17, 422–435 (1997). 9376117 10.1165/ajrcmb.17.4.2714

[b32] Hines W. C., Su Y., Kuhn I., Polyak K. & Bissell M. J. Sorting out the FACS: a devil in the details. Cell Rep. 6, 779–781 (2014). 24630040 10.1016/j.celrep.2014.02.021

[b33] Hammond S. L., Ham R. G. & Stampfer M. R. Serum-free growth of human mammary epithelial cells: rapid clonal growth in defined medium and extended serial passage with pituitary extract. Proc. Natl Acad. Sci. USA 81, 5435–5439 (1984). 6591199 10.1073/pnas.81.17.5435 PMC391719

[b34] Garbe J. C. . Molecular distinctions between stasis and telomere attrition senescence barriers shown by long-term culture of normal human mammary epithelial cells. Cancer Res. 69, 7557–7568 (2009). 19773443 10.1158/0008-5472.CAN-09-0270 PMC2782785

[b35] Zufferey R. . Self-inactivating lentivirus vector for safe and efficient *in vivo* gene delivery. J. Virol. 72, 9873–9880 (1998). 9811723 10.1128/jvi.72.12.9873-9880.1998 PMC110499

[b36] Barde I., Salmon P. & Trono D. Production and titration of lentiviral vectors. Curr. Protoc. Neurosci. Chapter 4, Unit 4 21 (2010). 20938923 10.1002/0471142301.ns0421s53

[b37] Sena-Esteves M., Tebbets J. C., Steffens S., Crombleholme T. & Flake A. W. Optimized large-scale production of high titer lentivirus vector pseudotypes. J. Virol. Methods 122, 131–139 (2004). 15542136 10.1016/j.jviromet.2004.08.017

[b38] Jeffers S. A., Sanders D. A. & Sanchez A. Covalent modifications of the ebola virus glycoprotein. J. Virol. 76, 12463–12472 (2002). 12438572 10.1128/JVI.76.24.12463-12472.2002 PMC136726

[b39] Kumar M., Bradow B. P. & Zimmerberg J. Large-scale production of pseudotyped lentiviral vectors using baculovirus GP64. Hum. Gene. Ther. 14, 67–77 (2003). 12573060 10.1089/10430340360464723

[b40] Presley J. F. . ER-to-Golgi transport visualized in living cells. Nature 389, 81–85 (1997). 9288971 10.1038/38001

[b41] Coil D. A. & Miller A. D. Phosphatidylserine is not the cell surface receptor for vesicular stomatitis virus. J. Virol. 78, 10920–10926 (2004). 15452212 10.1128/JVI.78.20.10920-10926.2004 PMC521854

[b42] Bieche I., Franc B., Vidaud D., Vidaud M. & Lidereau R. Analyses of MYC, ERBB2, and CCND1 genes in benign and malignant thyroid follicular cell tumors by real-time polymerase chain reaction. Thyroid 11, 147–152 (2001). 11288983 10.1089/105072501300042802

