# Supplementary material for: Modelling breast cancer requires identification and correction of a critical cell lineage-dependent transduction bias
Source: Nat Commun. 2015 Apr 21;6:6927. doi: 10.1038/ncomms7927 (PMC4411288; doi:10.1038/ncomms7927)
Supplement: Supplementary Information — Supplementary Figures 1-11, Supplementary Tables 1-4, Supplementary Notes 1-2, Supplementary Methods and Supplementary References [file ncomms7927-s1.pdf]

Supplementary Figures

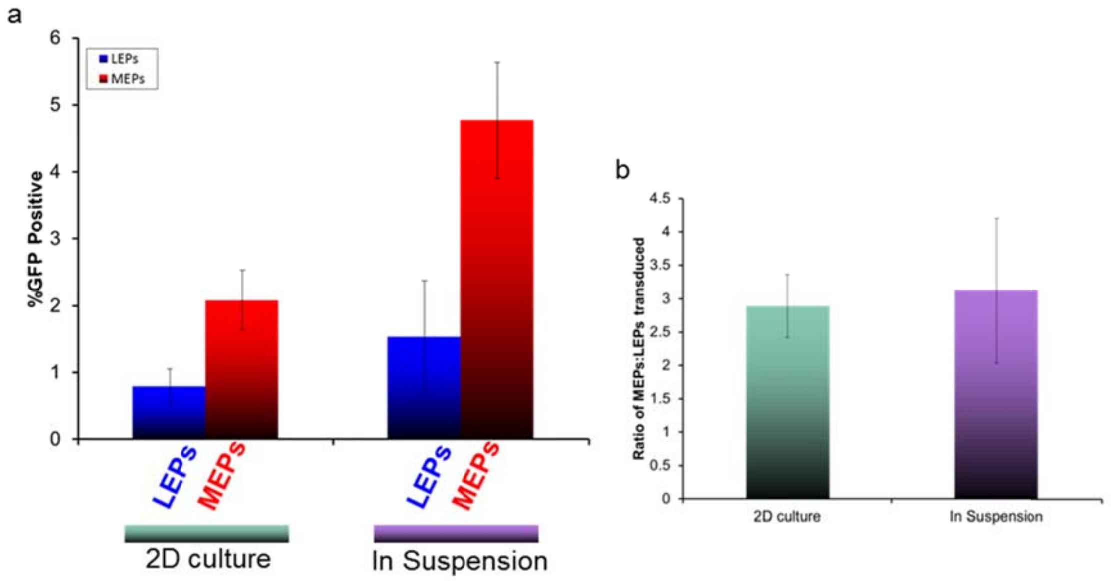

Supplementary Figure 1: Lentiviral transduction of primary cultures favors myoepithelial cells. Primary cells (passage 1), established from reduction mammoplasty tissue derived from a 17-year-old woman (sample N17), inoculated overnight with H2b-GFP encoding virus either as: 1) a standard 2-dimensional culture (turquoise) or 2) in suspension (purple). (a) Fraction of transduced primary LEPs (blue) and MEPs (red), as measured by flow cytometry (b) Ratio of MEPs to LEPs transduced. (mean ±S.D., n=3). We advise against suspension treatments because primary cells require contact to maintain their differentiated phenotype.

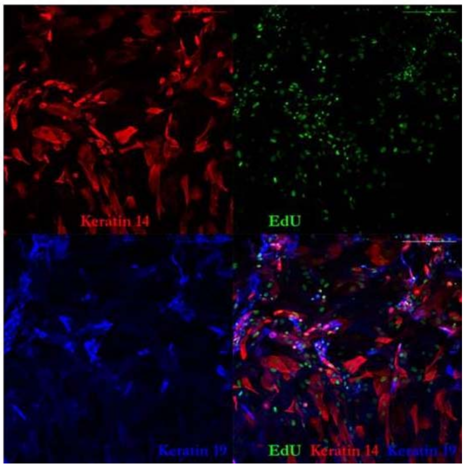

Supplementary Figure 2: Mitotic activity in LEP and MEP subpopulations. Incorporation of the nucleotide analog 5-ethynl-2'-deoxyuridine (EdU) in primary cells, cultured from reduction mammoplasty tissue of a 20-year-old woman. Cells were incubated for 48 hours with 1mM EdU and triple-stained for keratin 14 (red), and keratin 19 (blue) and EdU (green). Scale bars = 200µm.

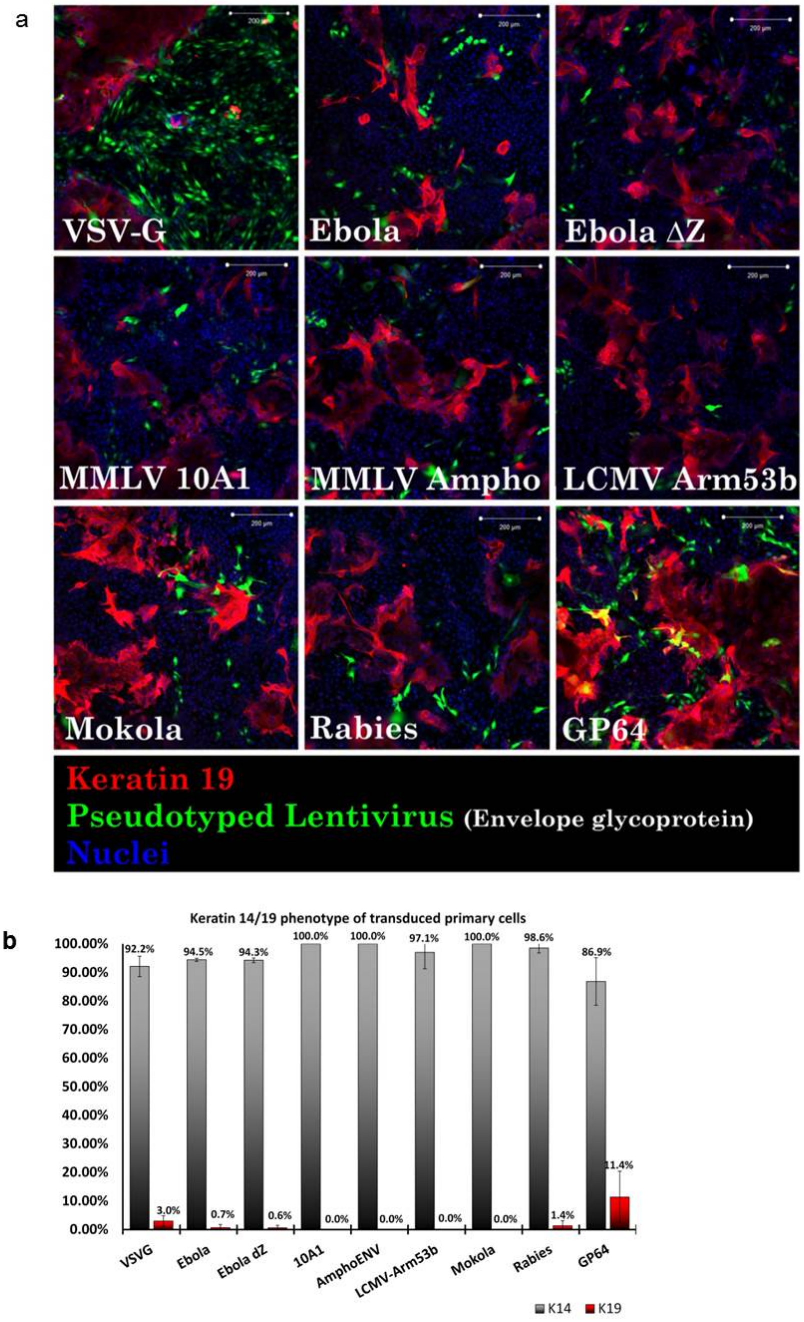

Supplementary Figure 3: The transductional bias exists even when using alternative lentiviral pseudotypes. (a) pseudotyped pLenti6/CMV-ZsGreen lentivirus particles were prepared as described in Methods using glycoproteins from the indicated viral species (white text). First passage primary cell cultures, derived from reduction mammoplasty tissue of a 34-year-old woman, were inoculated with each pseudotyped lentivirus, then co-stained for keratin 19 (red) and keratin 14 (not shown), as in Figure 1c. Scale bars = 200µm. (b) MEPs consistently constituted 85-100% of the transduced cells. (mean±SD, n=3)

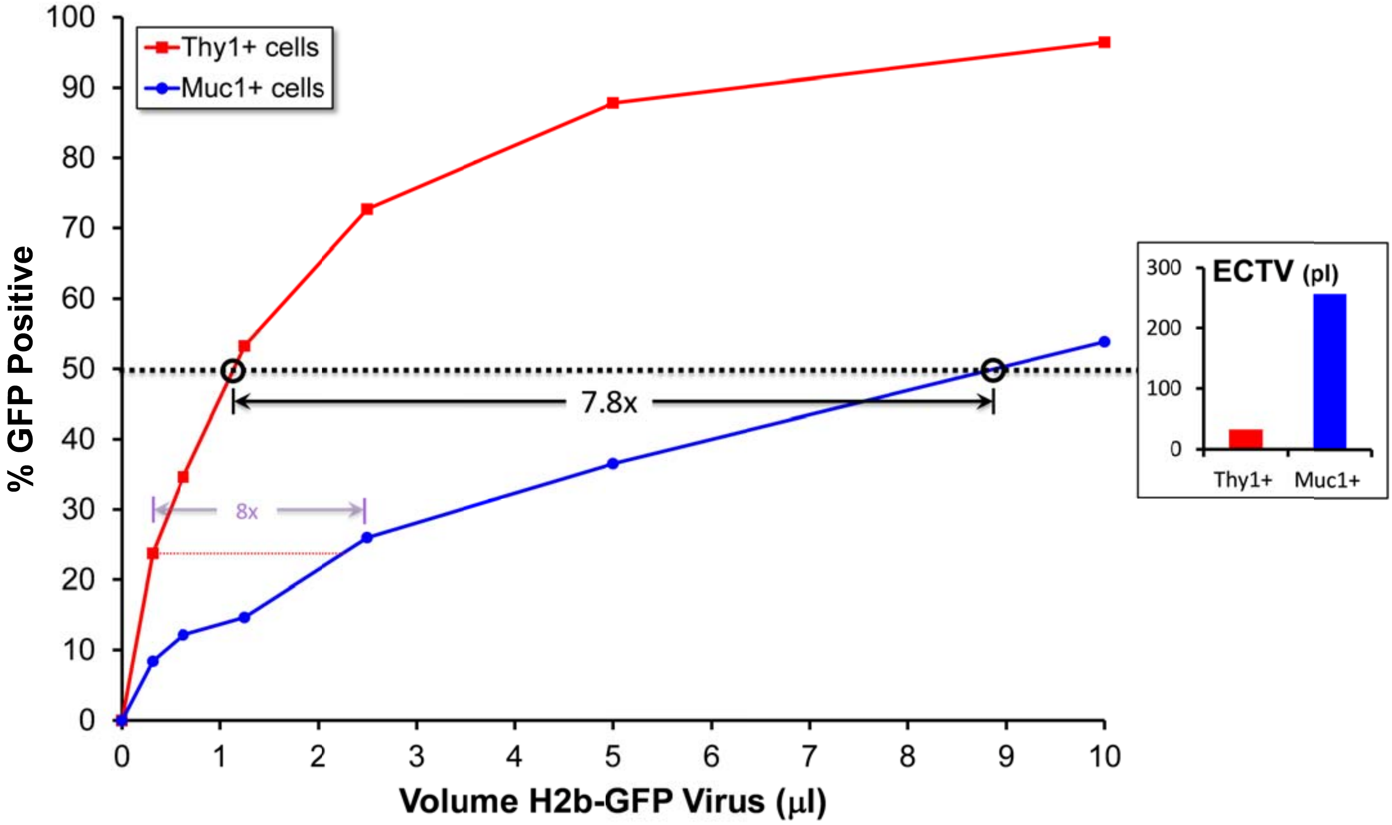

Supplementary Figure 4: Measuring transductional bias through ECTVs. ECTV values for the transduced primary MEP and LEP subpopulations, shown in Figure 1g, were calculated to be 32.9 and 256.5 picoliters, respectively (*inset, on right*). These values were ascertained using the equation given in Supplementary Note 1 by first determining the amount of virus required by each cell type to achieve a 50% transduction efficiency (dotted horizontal line); i.e, 1.14 µl (MEPs, red) and 8.88µl (LEPs, blue). The ratio of these ECTVs, 7.8x, is equivalent to the fold difference in viral volumes required to obtain equal level of transduction for the MEPs and LEPs, which can be also calculated at different levels of transduction; e.g., at 24% –the transduction efficiency obtained by MEPs at the lowest viral dilution (dotted red line). Each viral dilution is 2-fold, every third is 8-fold (*purple arrows*).

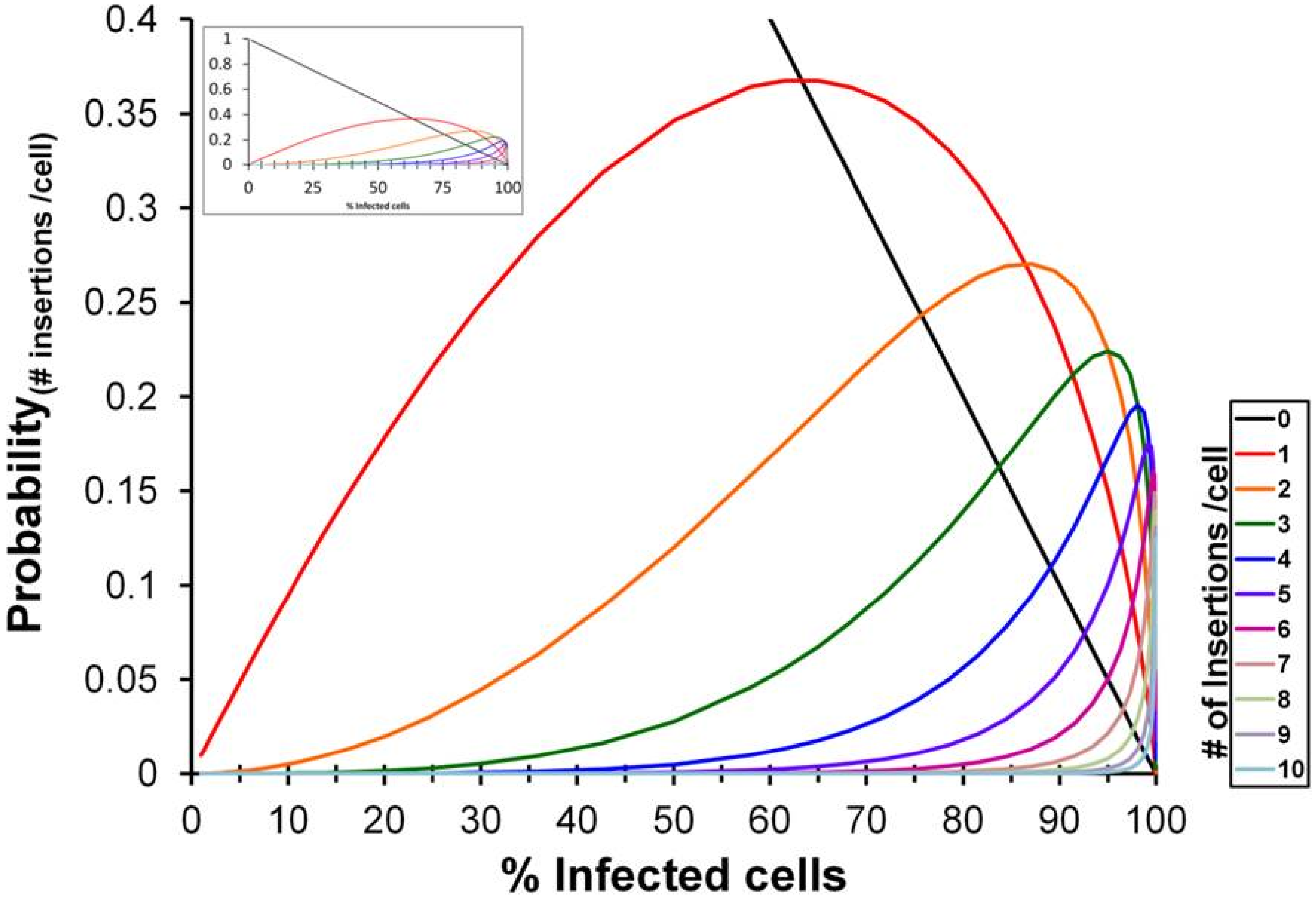

Supplementary Figure 5: Transduction efficiency and acquisition of multiple viral integrations, I. The Poisson distribution is useful in predicting viral transductions (supplementary note 2). Illustrated here is the theoretical relationship between the probable fractions of cells containing 0-10 integration events-per-cell (black, red, orange... traces) to the overall transduction efficiency (percentage of cells with at least one integration event; e.g., GFP^+). For example, when 50% of cells are transduced, the predicted fraction of cells with either 1, 2, or 3 integrations per cell are respectively 35, 12, and 3%, which is 70% (35/50), 24% (12/50) and 6% (3/50) of the ***transduced*** cell population. Inset: graph with y-axis expanded to 100%.

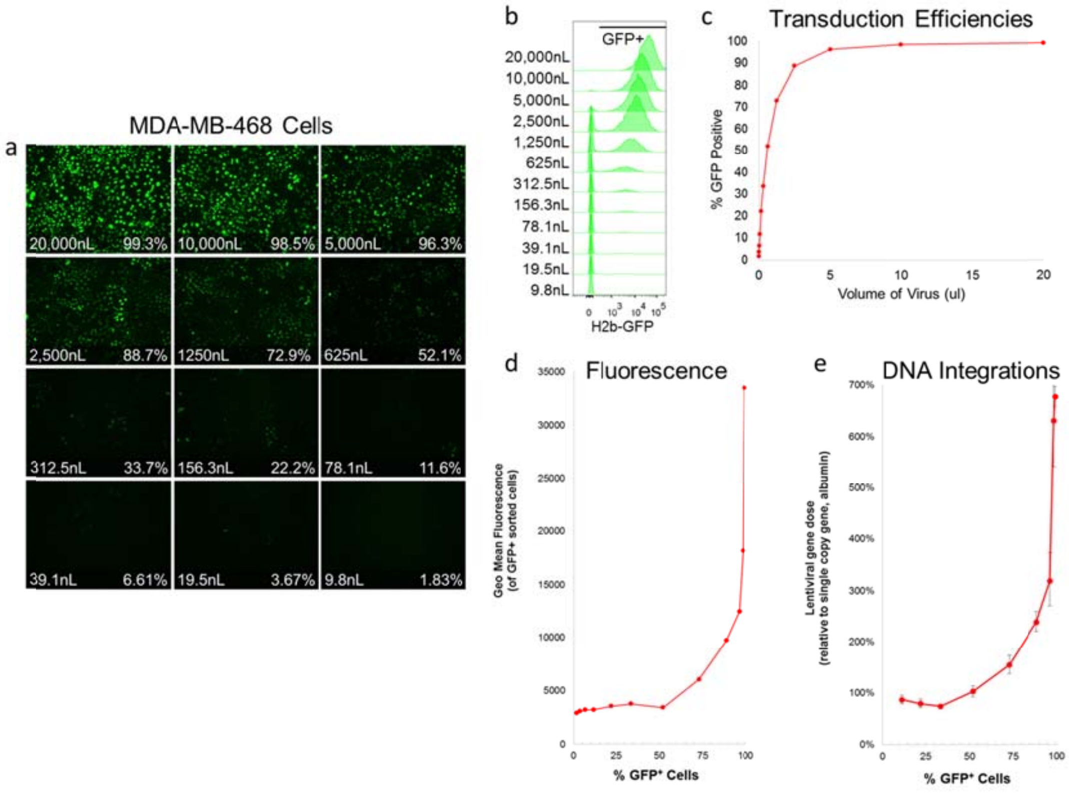

Supplementary Figure 6: Transduction efficiency and acquisition of multiple viral integrations, II. (a) To explore the relationship between transduction efficiency and lentiviral integrations (explained in part I, immediately above), MDA-MB-468 cells were transduced with 2-fold serial dilutions of CMV-H2b-GFP lentivirus and imaged by fluorescence microscopy; volumes of virus and the fractions of transduced cells that resulted from each infection are indicated. (b) Flow cytometry histograms, showing the level and distribution of GFP^+ cells (c) Graph of transduction efficiencies. (d,e) Fraction of cells (% GFP) that became transduced at each viral dose compared to (d) GFP fluorescence and (e) average lentiviral integration events. Lentiviral integrations were measured by qPCR and values are normalized to the single copy gene, albumin, (median±SD, n=4).

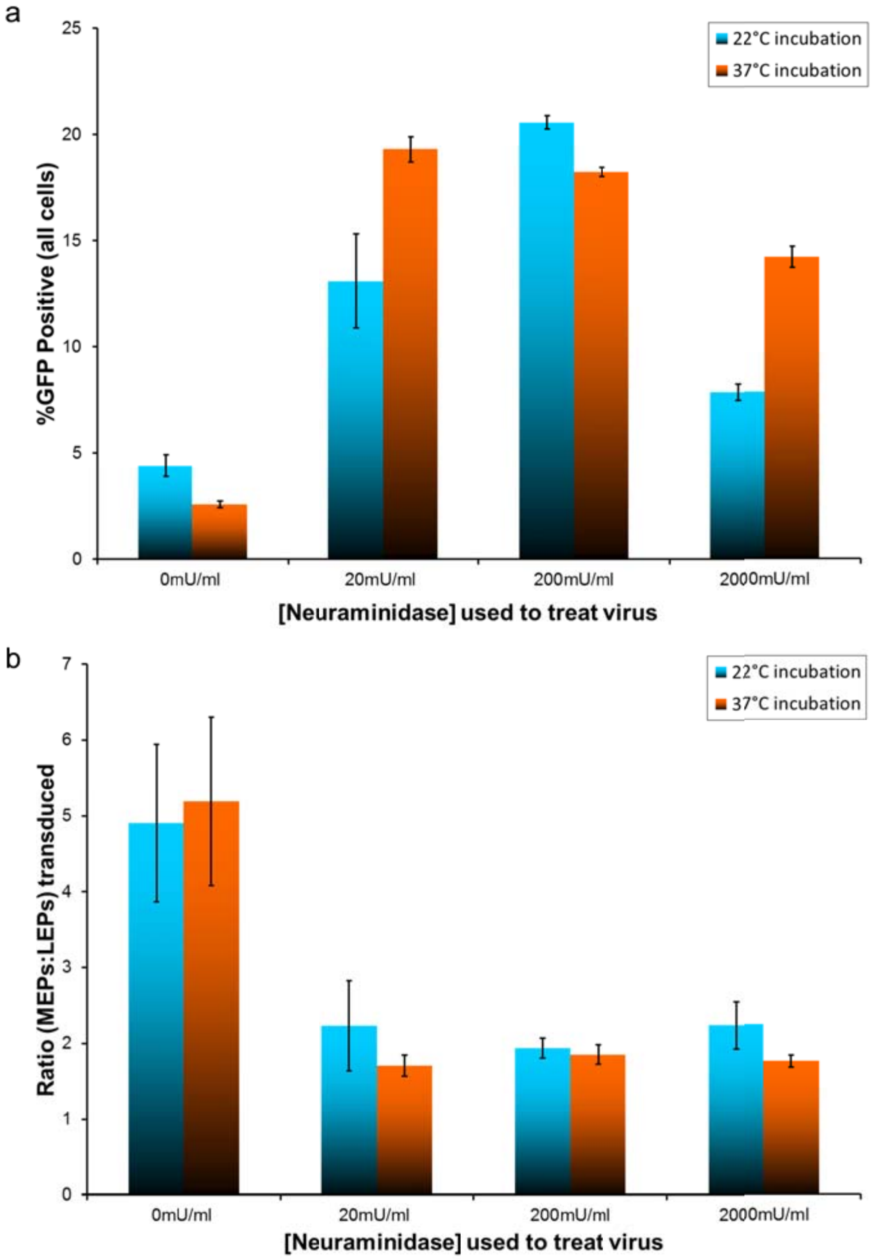

Supplementary Figure 7: Treating virus with neuraminidase improves viral efficiency and reduces myoepithelial to luminal bias. A concentrated preparation of H2b-GFP lentivirus was divided evenly and incubated with either: 0, 20, 200 or 2000mU/ml neuraminidase for 45 minutes at either 22°C (room temperature) or 37°C. These viruses were subsequently used to inoculate parallel primary (1p) cultures derived from a RMT from a 41-year-old woman (sample N156). After three days, the cells were dissociated, stained with Muc1 and Thy1 antibodies, and analyzed by flow cytometry to determine a) overall transduction efficiency and b) Ratio of MEPs:LEPs transduced. (mean±SD, n=3)

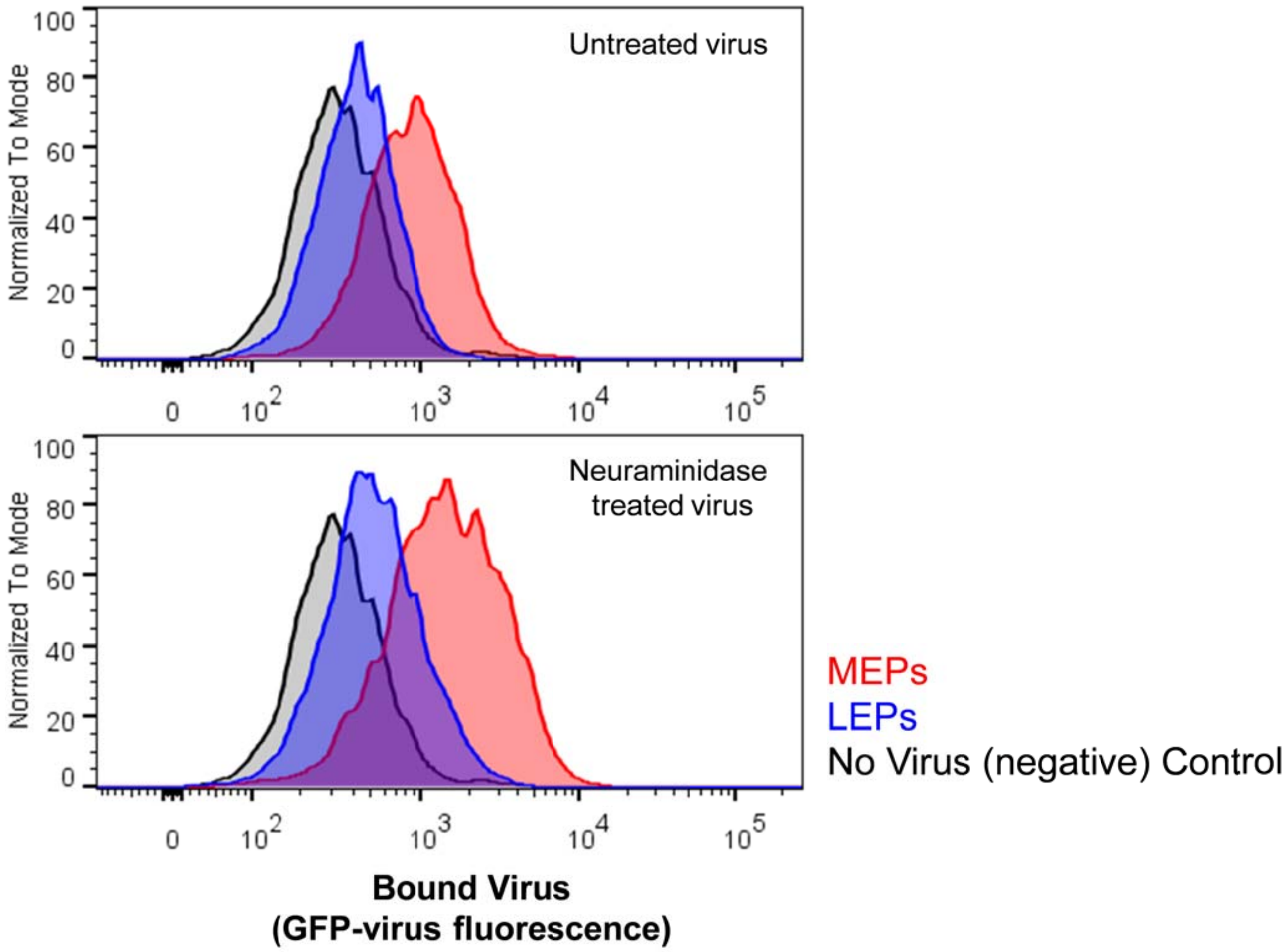

Supplementary Figure 8: Viral neuraminidase pretreatment improves affinity to primary breast cells. Viral affinity to primary (1st passage) cells was determined by measuring the amount of GFP retained on cells after briefly incubating them with fluorescent GFP-VSVG tagged lentiviral particles. A primary culture derived from a 41 y/o woman (sample N156) was dissociated and mixed in suspension with either untreated virus (*top*) or virus pre-treated with 200mU/ml neuraminidase (*bottom*). The cells were rinsed, stained with Muc1 and Thy1 antibodies, and analyzed by flow cytometry. The amount of cell bound virus (GFP fluorescence) measured in the luminal (LEPs, blue) and myoepithelial cell (MEPs, red) fractions is indicated. Black trace (*top & bottom*) = 'Fluorescence minus one' (no gfp-virus) negative control.

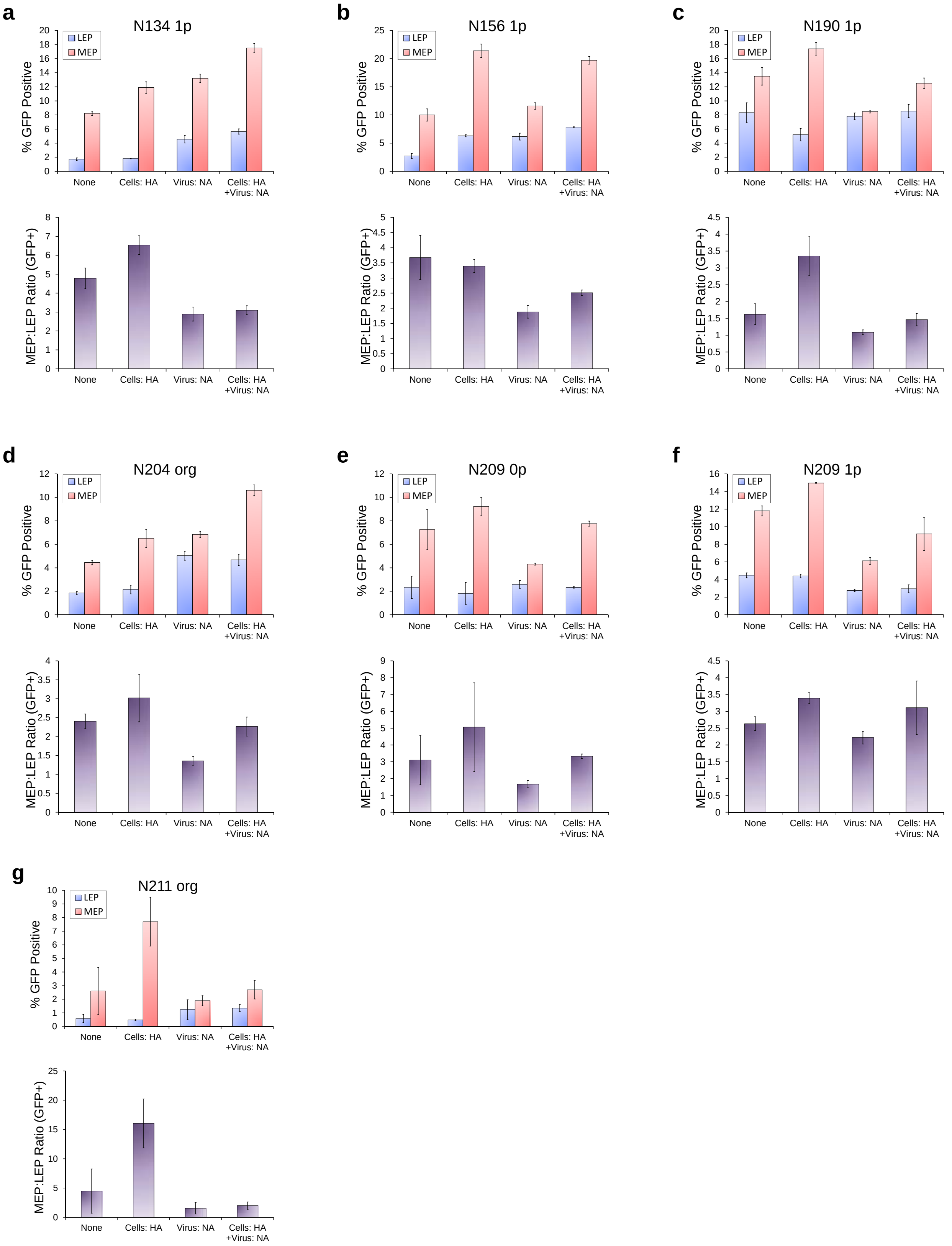

Supplementary Figure 9: Singular and combined effects of enzymatic pre-treatments to the transduction of primary breast cells. Seven primary cultures from organoids (org), dissociated organoids (0p), or 1st passage cells (1p) were established from 6 different individuals and transduced with lentivirus. The individual and combined effects of either 1) pretreating the cells with 2mg/ml hyaluronidase (37°C x 4 hours) and/or 2) pretreating viral stocks with 200mU/ml

neuraminidase (37°C x 45 min) were tested. Transduction efficiencies (%GFP Positive) of primary MEPs (*red*) and LEPs (*blue*) were determined by flow cytometry (*top graphs*). The calculated fractional difference (*purple*) between transduced MEPs and LEPs (MEP:LEP Ratio) is presented (*bottom graphs*) for each of the following conditions: untreated cells and virus (None), hyaluronidase treated cells (HA), virus treated with neuraminidase (NA) and the combination of both cell HA and virus NA treatments. (values=median±SD, n=3)

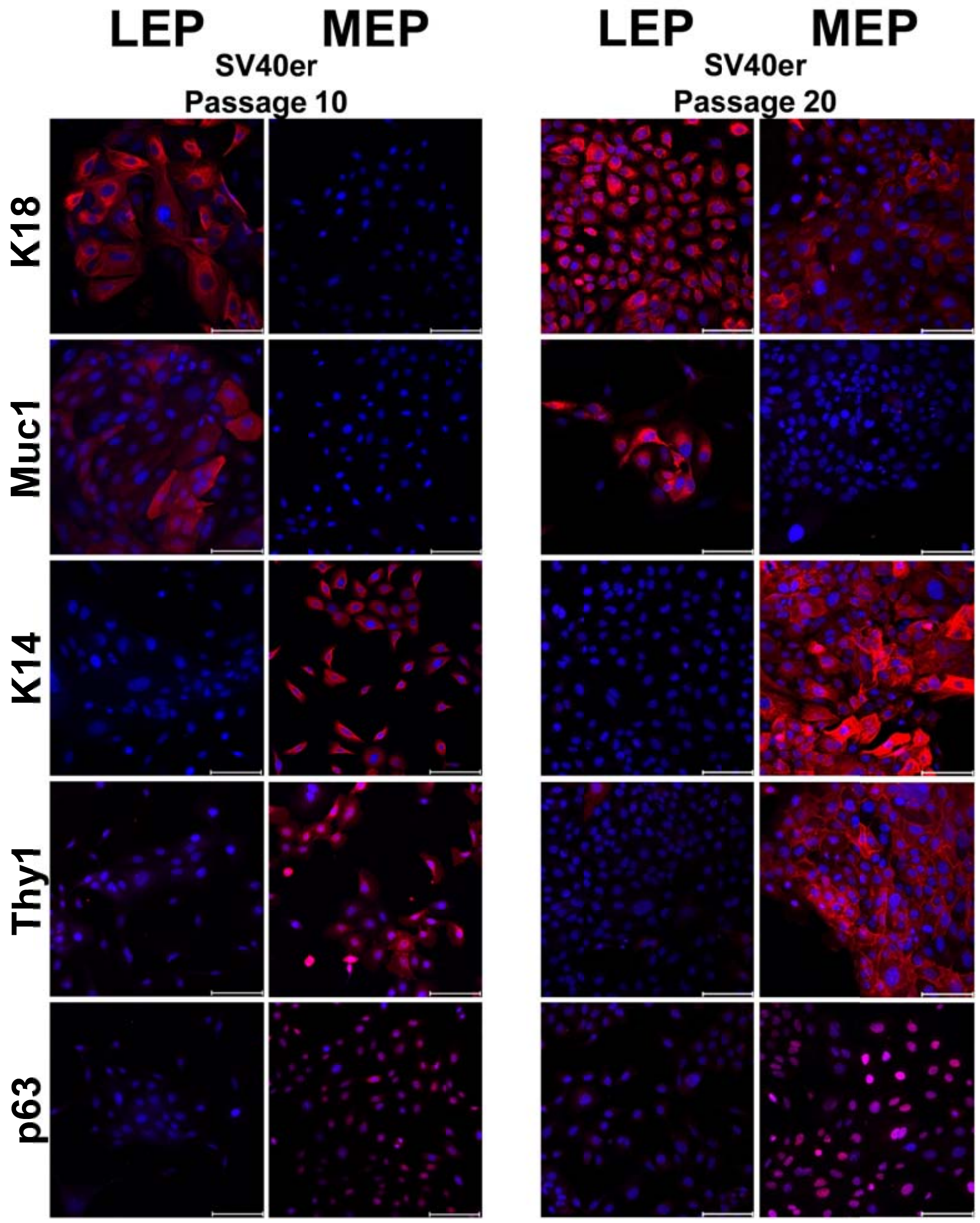

Supplementary Figure 10: Establishment of LEP and MEP cell lines with corresponding luminal and myoepithelial phenotypes. LEP and MEP cell lines generated from primary cultures treated with neuraminidase and transduced with lentivirus encoding SV40 early region (SV40 Large and Small T antigen). After transduction, LEP ($Muc1^+$) and MEP ($Thy1^+$) subpopulations were sorted and cultured separately until passage 20. Shown is 10th and 20th passage cells stained for markers of LEPs (K18 and Muc1) and MEPs (K14, Thy1, and p63). Scale bars = 100μm

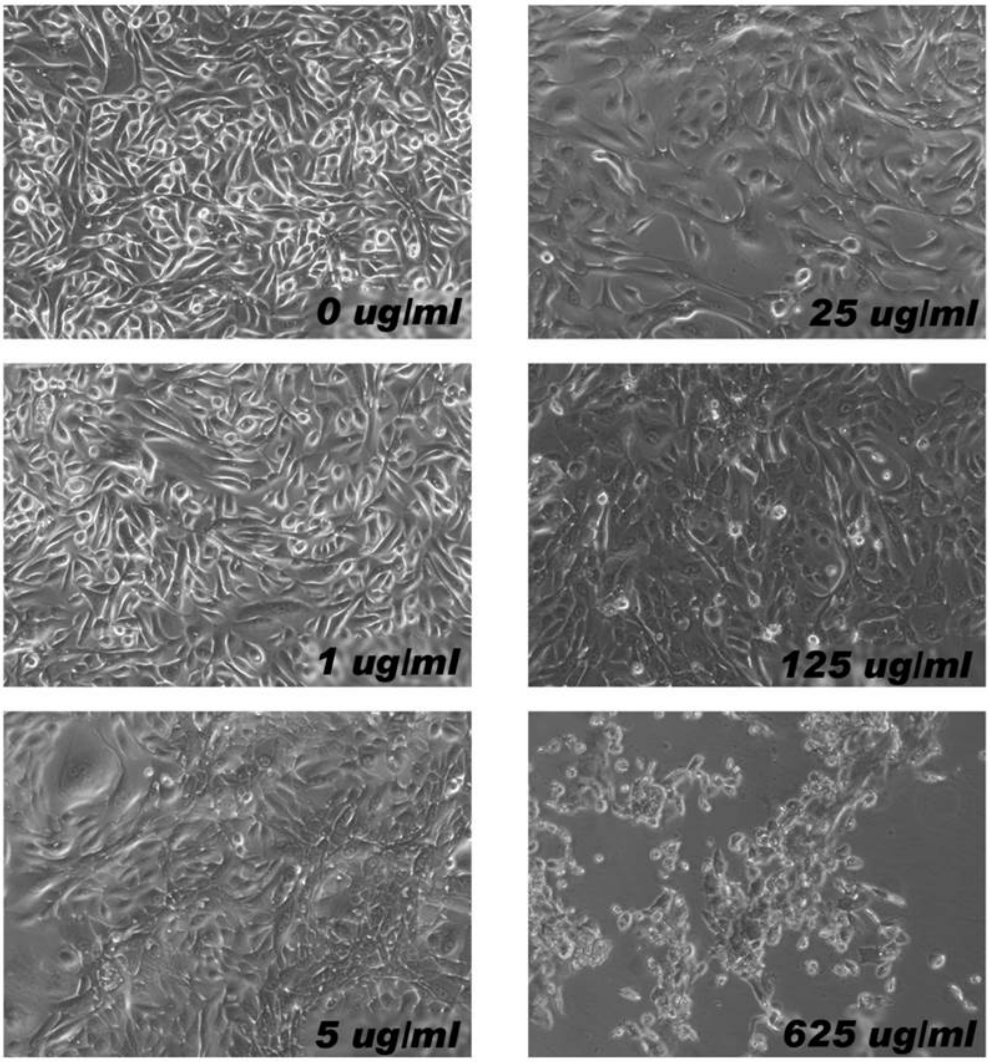

Supplementary Figure 11: Morphology of primary cells after polybrene exposure. Phase contrast images (100x) of primary cell outgrowths, derived from reduction mammoplasty tissue from a 48-year-old woman, cultured in MCDB170 medium, and incubated overnight with the indicated concentrations of polybrene (n=3). Stark morphological changes are evident in cells at 5μg/ml polybrene and above.

Supplementary Tables

Supplementary Table 1. Quantified primary LEP ($Muc1^+$) and MEP ($Thy1^+$) transductions

Sample[†]	%LEPs present	% $Muc1^+$ transduced	% $Thy1^+$ transduced	MEP:LEP % transduced Fold difference	MEP:LEP ECTV Fold difference
N17 (1p)	53%	3.32	13.2	3.98	4.19
N19 (1p)	33%	2.09	13.7	6.56	6.98
N27 (1p)	56%	4.15	22.5	5.42	6.01
N28 (0p)	29%	16.8	57.1	3.40	4.60
N116 (0p)	23%	7.6	17.5	2.30	2.43
N120 (0p)	52%	16.7	68.1	4.08	6.25
N134 (1p)[Δ]	52%	6.68	26	3.89	4.36
N135 (0p)[§]	31%	4.3	64.3	14.95	23.44
N135 (1p)[§]	10.1%	14.7	53.3	3.63	7.79
N138 (0p)	30%	2.6	15.9	6.12	6.57
N138 1p[§]	10.3%	7.4	25	3.38	2.91
FACS Sorted:					
N120 (1p)[‡] LEPs	100%	10.8	N/A	3.3	3.9
N120 (1p)[‡] MEPs	0%	N/A	36.0		

Percentages of transduced cells as measured by flow cytometry. [†]calculated from independent experiments without neuraminidase or hyaluronidase treatments. [Δ]6µg/ml polybrene used. [‡] inoculated as autonomous (FACS sorted) cultures. [§]These data also included in manuscript figures.

Supplementary Table 2. ATCC Cell lines

Cell Line	ATCC designation	Passage Number	Culture Medium
MDA-MB-231	HTB-26	28p	DMEM, 10%FBS
HCC38	CRL-2314	ATCC: unknown passage	RPMI, 10%FBS
BT549	HTB-122	40p	RPMI, 10%FBS
T47D	HTB-133	88p	RPMI, 10%FBS
HCC1428	CRL-2327	30p	RPMI, 10%FBS
AU565	CRL-2351	103p	RPMI, 10%FBS
MCF-7	HTB-22	148p	DMEM, 10%FBS
MDA-MB-468	HTB-132	339p	DMEM, 10%FBS
HCC1937	CRL-2336	17p	RPMI, 10%FBS
HCC1954	CRL-2338	22p	RPMI, 10%FBS

Cell lines, ATCC designation, Acquired passage number and culture conditions. Cells were cultured at 37°C in 5% CO_2.

Supplementary Table 3. Antibodies

**Antibody specificity**	**Clone**	**Conjugate**	**Source**
α6 Integrin (CD49f)	GoH3	PE	BioLegend
c-Kit (CD117)	104D2	PE/Cy7	BioLegend
EpCam (CD326)	9C4	APC	BioLegend
CALLA (CD10)	HI10a	APC/Cy7	BD Biosciences
Muc1 (CD227)	HMPV	FITC and unlabeled	BD Biosciences
Thy1 (CD90)	5E10	PE/Cy5	BD Biosciences
Keratin 14	Rabbit polyclonal (Rb-9020)	None	Neomarkers/Thermo
Keratin 19	A53-B/A2.26	None	Neomarkers/Thermo
Keratin 18	DC10	None	Neomarkers/Thermo
P63	4A4	None	abcam

Antibodies used for flow cytometry, FACS, and immunocytochemistry.

Supplementary Table 4. PCR Primers used for lentivirus cloning

Amplicon	Template Plasmid	Primer Name	Sequence*	PCR product
EGFP	EGFP-N1	EGFP-1 TOPO FOR	CACC**ATG**GTGAGCAAGGGCGA	730bp
		EGFP-1 TOPO REV	GCCGCT**TTA**CTTGTACAGCTCGTC	
ZsGreen	pZsGreen1-DR	ZsGreen-DR D-TOPO FOR	CACC**ATG**GCCCAGTCCAA	700bp
		ZsGreen D-TOPO REV v.2	**TTA**GGG CAA GGC GGA GCC	
H2B-GFP	H2B-GFP	H2B-GFP FOR	CACC**ATG**CCAGAGCCAGCGAA	1126bp
		H2B-GFP REV	GCCGCT**TTA**CTTGTACAGCTCGTCCAT	
mCherry	pmCherry-ATG5	EGFP-1 TOPO FOR	CACC**ATG**GTGAGCAAGGGCGA	
		mCherry D-TOPO REV	**TTA**CTTGTACAGCTCGTCCATGCCG	
CMV	pShuttle HA-BORIS	CMV IE promoter FOR	GGAGTTCCGCGTTACATAACTTACGGT	529bp
		CMV IE promoter REV	AGTTAGCCAGAGAGCTCTGCTTAT	

Contained are the primer sequences used for lentivirus subcloning. *start (*green*) and stop codons (*red*) are underlined in bold text.

Supplementary Notes

Supplementary Note 1. The Effective Cell Transducing Volume

In their seminal article on culturing poliovirus, Renato Dulbecco and Marguerite Vogt[1] used the Poisson distribution to predict the fraction of virally infected cells in culture that would result from inoculating a given number of cells (*N*) with a limiting dilution of poliomyelitis virus, by:

$$\frac{Plaques}{N} = 1 - e^{-(\frac{a*v}{N})}$$

where (v) is the number of virus particles and (a) is defined as ‘a constant defining the efficiency of the system,’[1]. Thus, $(a * v)$ becomes the number of *effective* infectious units, and $(a * v)/N$ is equivalent to the effective infectious units per cell. In an infection where the effective infectious units per cell is equal to one (one particle per cell in a 100% efficient system), the estimated fraction of infected cells calculates to be 63.2% (i.e., $1 - e^{-1}$). Tables of the values derived from the Poisson distribution have been published and discussed elsewhere; for example, see references [2,3]). Here, we reasoned that the number of effective infectious units (live virus particles capable of transducing a cell) is difficult, if not impossible, to precisely quantify; however, the *volumes* of virus are both precisely and easily determined. Thus, if we reinterpret the efficiency, (‘a’), in terms of volume of virus used, rather than the number of viral particles, the probability of uninfected cells is simply:

$$P_{(0)} = \mathrm{e}^{-\left(\frac{Vol}{N*ECTV}\right)}$$

In this equation, $P_{(0)}$ is the probability or fraction of uninfected cells, *Vol* is the total volume of inoculum (concentrated viral stock) used, *N* is the number of cells. We define *ECTV* as the “effective cell transducing volume.” It is the specific volume of virus that is equivalent to a single 100% effective transduction unit when applied to a given homogeneous cell population under a specific set of experimental conditions (to gain a better understanding of the implications and applicability of the above expression, see supplementary note 2). ECTV is empirically calculated by determining the volume of inoculum needed to infect 50% of cells in a culture under a *specific* set of experimental conditions (Vol_{50}):

$$ECTV = \frac{Vol_{50}}{(N)} * \frac{1}{0.693}$$

For cases when the fraction of infected cells does not approach 50%, ECTV can similarly be calculated for a smaller percentage of infected cells, as long as the number of cells transduced is sufficiently above background levels.

$$ECTV = \frac{Vol_{X\%}}{-N(\ln(1 - \frac{x\%}{100}))}$$

Once ECTV is calculated for a type of cell, virus preparation and set of conditions, the volume of virus needed to infect a specific fraction of cells in subsequent experiments, using identical experimental conditions (e.g., see Figure 3), is given by:

$$Vol_{y\%} = -ECTV(N)\ln(1 - \frac{y\%}{100})$$

Supplementary Note 2. Poisson Distribution for viral transduction

If one introduces a number of lentivirus particles to a large number of cells, the Poisson distribution describes the probability, P(x), of any given cell receiving 'x' virus particles, where lambda (λ) is the average number of virus particles per cell. If the virus particles are 100% effective, P(x) is also the probability of 'x' transductions per cell.

$$P_{(x)} = \frac{\lambda^x \cdot e^{-\lambda}}{x!}$$

We will show here that P(x) can also be calculated by:

$$P_{(x)} = \frac{{\lambda_v}^x \cdot e^{-\lambda_v}}{x!}$$

Where, 'λ_v' is the effective viral dose (i.e., Vol/(N*ECTV), and 'x' is the number of transductions (viral integrations) a cell will receive. Thus, using the Poisson distribution with these substitutions treats a cell not receiving an ECTV as not transduced and a cell receiving three ECTV's as triply transduced (i.e., having three integration events). This treats a volume as an indivisible unit, which makes little physical sense. However, this simple result can be shown to be accurate when a particle-based analysis is performed.

It is important to distinguish here between the concept of a '100% effective virus particle' and actual physical virus particles, a single one of which may not successfully transduce a cell. For the sake of clarity, actual physical virus particles will be referred to here as 'virions.' Therefore, each virion will have some probability, p, of transducing the cell, and p could be quite small. In this case, the probability of the number of viral transductions a cell suffers, 'i,' given exposure to 'x' virions, is given by the binomial distribution, where the probability of failure, 'q', is simply '1-p':

$$P_{(i,x)} = \frac{x!}{i! \cdot (x-i)!} \cdot p^i \cdot q^{(x-i)}$$

Combining this probability with the above probability of the number of exposures, 'x':

$$P_{(i)} = \sum_{x=i}^{\infty} \frac{\lambda^x \cdot e^{-\lambda}}{x!} \cdot \frac{x!}{i! \cdot (x-i)!} \cdot p^i \cdot q^{(x-i)}$$

Here, the lower limit starts at 'i' because there is no chance of 'i' infections if there are not at least 'i' virions introduced. The upper limit of infinity simply approximates the total number of virions available to all cells. Simplifying and pulling out all non-x dependent terms:

Since $\lambda^x = \lambda^i \cdot \lambda^{(x-i)}$

$$P_{(i)} = \frac{\lambda^i \cdot e^{-\lambda} \cdot p^i}{i!} \cdot \sum_{x=i}^{\infty} \frac{(\lambda \cdot q)^{(x-i)}}{(x-i)!}$$

With the understanding that the summation can only be carried out to the total number of virions introduced to all the cells, rather than infinity, the summation is the Maclaurin series expansion for $e^{\lambda q}$. Therefore:

$$P_{(i)} = \frac{\lambda^i \cdot p^i}{i!} \cdot e^{-\lambda \cdot (1-q)}$$

Because (1-q) is simply ‘p’:

$$P_{(i)} = \frac{(\lambda \cdot p)^i \cdot e^{-\lambda \cdot p}}{i!}$$

So, does $\lambda \cdot p$ equal λ_v?

If D is the density of virions in the medium bathing the cells, then:

$$\lambda = D \cdot \frac{Vol}{N}$$

What remains is to determine p in terms of ECTV. From the definition of ECTV (equation #2, Supplementary Note 1), if $Vol/N =$ ECTV, then the probability of any cell to not be transduced, P(0), is simply equal to e^{-1}

Stated in terms of virions,

$$(1-p)^{D \cdot ECTV} = e^{-1}$$

Then,

$$D \cdot ECTV \cdot ln(1-p) = -1$$

Therefore,

$$p = 1 - e^{-\left(\frac{1}{D \cdot ECTV}\right)}$$

Expanding p in a Maclaurin series,

$$p = \frac{1}{D \cdot ECTV} - \frac{1}{2} \cdot \frac{1}{(D \cdot ECTV)^2} + \frac{1}{6} \cdot \frac{1}{(D \cdot ECTV)^3} - \ \dots$$

Thus, if the number of virions contained in an ECTV, ‘$D \cdot ECTV$’, is much larger than one, then this simplifies to:

$$p = \frac{1}{D \cdot ECTV}$$

And finally, we see that $\lambda \cdot p$ does in fact equal λ_v:

$$\lambda \cdot p = \frac{Vol}{N \cdot ECTV} \quad (which\ is\ \lambda_v)$$

Thus, the assumption that a volume, the *Effective Cell Transducing Volume* (ECTV), can be treated as an indivisible unit obeying a Poisson distribution is, in fact, a very accurate approximation for small p.

Supplementary Methods

Lentiviral constructs and detailed cloning information

Lentiviral transfer vectors were constructed using the modular MultiSite Gateway® cloning technology (Virapower promoterless lentiviral gateway system, Invitrogen) by LR recombination reactions containing three plasmids: 1) the pLENTI6/R4R2/V5 destination vector, 2) a L4R1 promoter entry plamid (pENTR5'UbCp or pENTR5'CMV), and 3) a L1/L2 gene entry clone (either pENTRD-EGFP, pENTRD-ZsGreen, pENTRD-H2b-GFP, pENTRD-mCherry, or PENTR1A-SV40er (addgene 22297). The resulting lentiviral vectors (pLenti6/ UbC-EGFP, pLenti6/CMV-ZsGreen, pLenti6/CMV-H2B-GFP, and pLenti6/UbC-mCherry) were all confirmed by restriction mapping, DNA sequencing, and functionally characterized by fluorescent microscopy and flow cytometry. To create the promoter entry vecors, cytomegalovirus (CMV_{IE}) promoter sequence derived from pShuttle-BORIS[4] was PCR cloned into pENTR5'-TOPO attL4/R1 plasmid (Invitrogen), whereas the human UbC promoter construct (pENTR5'UbCp) was provided by Invitrogen. The gene entry clones (pENTRD-EGFP, pENTRD-ZsGreen, pENTRD-mCherry, and pENTRD-H2b-GFP) encoding the fluorescent proteins EGFP, ZsGreen, mCherry, and a fusion protein of human histone H2B and GFP (H2B-GFP), were created by cloning the respective PCR products of the coding regions into the attL1/L2 pENTR-D TOPO plasmid (Invitrogen). Templates for these reactions were provided by the pEGFP-N1and pZsGreenDR plasmids (Clontech), whereas the mCherry and H2B-GFP fragment were respectively amplified from pmCherry ATG5 (Addgene plasmid 13095) and H2B-GFP (Addgene plasmid 11680), kindly submitted to Addgene by Roberta Gottlieb and Geoff Wahl. pENTR SV40 small and large T in pENTR1A was provided by Eric Campeau (Addgene 22297). PCR primers used to create these constructs are in Supplementary Table 4:

Supplementary References

1. Dulbecco, R. & Vogt, M. Plaque formation and isolation of pure lines with poliomyelitis viruses. *The Journal of experimental medicine* **99**, 167-182 (1954).
2. Fehse, B., Kustikova, O.S., Bubenheim, M. & Baum, C. Pois(s)on--it's a question of dose. *Gene therapy* **11**, 879-881 (2004).
3. Knipe, D. & Howley, P. *Fields virology. vol. 1*, (Lippincott Williams & Wilkins, Pa., USA, 2006).
4. Hines, W.C., Bazarov, A.V., Mukhopadhyay, R. & Yaswen, P. BORIS (CTCFL) is not expressed in most human breast cell lines and high grade breast carcinomas. *PLoS One* **5**, e9738 (2010).
